# PrP aggregation can be seeded by pre-formed recombinant PrP amyloid fibrils without the replication of infectious prions

**DOI:** 10.1007/s00401-016-1594-5

**Published:** 2016-07-04

**Authors:** Rona M. Barron, Declan King, Martin Jeffrey, Gillian McGovern, Sonya Agarwal, Andrew C. Gill, Pedro Piccardo

**Affiliations:** 1 The Roslin Institute and R(D)SVS, University of Edinburgh, Easter Bush, Midlothian, EH25 9RG Scotland, UK; 2 Animal and Plant Health Agency, Pentlands Science Park, Midlothian, Scotland, UK

**Keywords:** Amyloid, Seeding, PrP, Prion, TSE

## Abstract

Mammalian prions are unusual infectious agents, as they are thought to consist solely of aggregates of misfolded prion protein (PrP). Generation of synthetic prions, composed of recombinant PrP (recPrP) refolded into fibrils, has been utilised to address whether PrP aggregates are, indeed, infectious prions. In several reports, neurological disease similar to transmissible spongiform encephalopathy (TSE) has been described following inoculation and passage of various forms of fibrils in transgenic mice and hamsters. However, in studies described here, we show that inoculation of recPrP fibrils does not cause TSE disease, but, instead, seeds the formation of PrP amyloid plaques in PrP-P101L knock-in transgenic mice (101LL). Importantly, both WT-recPrP fibrils and 101L-recPrP fibrils can seed plaque formation, indicating that the fibrillar conformation, and not the primary sequence of PrP in the inoculum, is important in initiating seeding. No replication of infectious prions or TSE disease was observed following both primary inoculation and subsequent subpassage. These data, therefore, argue against recPrP fibrils being infectious prions and, instead, indicate that these pre-formed seeds are acting to accelerate the formation of PrP amyloid plaques in 101LL Tg mice. In addition, these data reproduce a phenotype which was previously observed in 101LL mice following inoculation with brain extract containing in vivo-generated PrP amyloid fibrils, which has not been shown for other synthetic prion models. These data are reminiscent of the “prion-like” spread of aggregated forms of the beta-amyloid peptide (Aβ), α-synuclein and tau observed following inoculation of transgenic mice with pre-formed seeds of each misfolded protein. Hence, even when the protein is PrP, misfolding and aggregation do not reproduce the full clinicopathological phenotype of disease. The initiation and spread of protein aggregation in transgenic mouse lines following inoculation with pre-formed fibrils may, therefore, more closely resemble a seeded proteinopathy than an infectious TSE disease.

**Electronic supplementary material:**

The online version of this article (doi:10.1007/s00401-016-1594-5) contains supplementary material, which is available to authorized users.

## Introduction

The misfolding and aggregation of host protein in the brain is a pathological characteristic of several neurodegenerative diseases, such as Alzheimer’s disease (AD), Parkinson’s disease (PD) and transmissible spongiform encephalopathy (TSE). However, the actual role of these protein aggregates in the neurodegenerative process is currently unclear. TSEs differ from other neurodegenerative diseases, since they affect several mammalian species other than humans and are infectious. TSEs such as scrapie in sheep and goats, bovine spongiform encephalopathy (BSE) in cattle and Creutzfeldt–Jakob disease (CJD) in humans can be transmitted between individuals of the same species and, in some cases, can spread between different species. TSEs exist as a large number of strains/isolates that show specific, reproducible clinical and pathological characteristics on transmission in animals. They can be contagious and can spread horizontally between animals in the field, such as scrapie in sheep and chronic wasting disease (CWD) in deer [60]. Other TSEs, such as BSE in cattle and CJD in humans, are not contagious, but are infectious, and can be transmitted indirectly between individuals following ingestion of contaminated tissues [3, 4, 19, 73], inoculation of such tissues into the CNS or periphery [1, 2, 7, 36], or transfusion of blood [26, 27, 38, 57, 74]. The infectious agent responsible for TSE is not a conventional pathogen such as a bacterium or virus. However, the disease is caused by a titratable infectious agent, which maintains specific characteristics on transmission in animals. It is thought that the infectious agent is a misfolded form of the host prion protein (PrP^C^) [59]. This misfolded protein propagates by binding to and converting PrP^C^ into the abnormal isoform (PrP^Sc^), and this autocatalytic conversion results in the deposition and spread of PrP^Sc^ through the central and peripheral nervous system, and also some viscera such as the lymphoreticular system, in patterns characteristic of each individual TSE strain/isolate.

The central role of the prion protein in TSE disease has led to the infectious agent being termed a prion [59] and the disease being referred to as prion disease. However, the term “prion” or “prion-like” has more recently been used to describe the observed spread of protein aggregates and amyloid accumulation in the brains of transgenic mice expressing either wild-type or mutant [51] human amyloid precursor protein (APP), following intracerebral [47, 49, 70, 71] and peripheral [17] inoculation with pre-formed aggregates of beta-amyloid peptide (Aβ). Similar models of induced protein aggregation have also been described for PD (inoculation of α-synuclein aggregates) [50], amyotrophic lateral sclerosis (ALS) (inoculation of misfolded SOD-1) [52] and tauopathy (inoculation of filamentous tau) [11]. “Prion-like” spread occurs when pre-formed protein aggregates are introduced into a host, which has the effect of accelerating the misfolding and aggregation of the endogenous host protein in the brain. On this basis, it is entirely possible that these pre-formed aggregates of Aβ, α-synuclein, SOD-1 and tau are acting as “prions” and are providing a template driving the misfolding of normal cellular forms of the protein into amyloid fibrils. However, in APP transgenic mouse models, acceleration of protein aggregation has been observed only following direct intracerebral inoculation [47, 50, 71] or peripheral inoculation [17]. Protein aggregation is not accelerated by oral, intravenous, intraocular or intranasal inoculation [15]. Several groups have demonstrated the potential for existence of “strains” of Aβ and α-synuclein [5, 25], and other reports described Aβ amyloid plaques in human growth hormone recipients [28, 29], and dura matter graft recipients [21, 35]. However, no definitive evidence of direct or indirect transmission has been described for either AD or PD in humans [28].

The generic use of the terms “prion” and “prion-like” to describe various aspects of all neurodegenerative diseases does, therefore, cause confusion. AD, PD, ALS and tauopathy are not transmissible diseases, and there is no epidemiological evidence to support an infectious aetiology [28]. These diseases are, therefore, distinctly different in their aetiology from TSEs. It has been proposed that the extended term “propagon” could be used to simply describe that a misfolded protein is being propagated, and avoid confusion over prion transmissibility and infection [16]. In this paper, we will use the terms “prion” and “prion-like” to describe the general mechanism of protein aggregation and spread, and not as a surrogate for TSE.

Although the TSE agent is thought to be PrP^Sc^, previous work from our laboratory has shown that PrP aggregation does not always lead to replication of an infectious agent and subsequent TSE [55, 56]. There exists a subset of cases in which PrP aggregates are formed in the brain in the absence of TSE agent replication [9, 10, 24, 55, 56]. One such disorder that exemplifies this phenomenon is a variant of Gerstmann–Sträussler–Scheinker (GSS) disease, a familial human TSE disease characterised by PrP amyloid plaque deposition. The most common form of GSS is associated with a proline-to-leucine mutation at PrP codon 102 (GSS P102L) [54]. Inoculation of knock-in transgenic mice homozygous for the equivalent mutation in murine PrP (101LL) with classical forms of GSS P102L (associated with spongiform degeneration and diffuse PrP deposition) resulted in the development of clinical and pathological signs of TSE disease. When 101LL mice were inoculated with atypical forms of GSS P102L (no spongiform change and PrP amyloid plaques), inefficient disease transmission was observed with most animals surviving for full lifespan with no clinical signs or spongiform degeneration. However, on postmortem analysis, many of the inoculated mice were found to have large PrP amyloid plaques in the brain [56]. Similarly, when 101LL mice were inoculated with brain homogenate from sick GSS22 mice [53] (which overexpress 101L murine PrP and spontaneously develop neurological signs and large PrP amyloid plaques), no clinical signs of TSE disease or spongiform degeneration were evident, but large PrP amyloid plaques were again identified in the brains of these mice postmortem [55]. Hence, extracts from both humans with an atypical form of GSS P102L and transgenic mice (GSS22) overexpressing murine 101L-PrP (both characterised by PrP amyloid plaque disposition) do not transmit TSE disease to 101LL mice. Components of the brain inoculum are instead capable of seeding the aggregation of PrP amyloid plaques in recipient mice expressing 101L but not wild-type PrP. However, disease may be transmissible from such atypical TSE isolates in other model systems [58].

Although the above data argue for a direct “seeding” of PrP amyloid deposition by pre-formed PrP aggregates in the inoculum, our experiments performed to date have utilised brain material harvested from individual patients or mice with neurological symptoms, which may contain other components, or possibly low levels of an infectious agent, in addition to pre-formed PrP amyloid seeds. Therefore, in this study, we aimed to investigate directly whether misfolded forms of PrP, in the absence of other components of brain homogenate, can seed amyloid plaque formation, or cause TSE disease, following intracerebral inoculation in recipient mice. To this end, wild-type murine recombinant PrP (WT-recPrP) and 101L murine recPrP (101L-recPrP) preparations were refolded into different conformations in vitro and inoculated into groups of wild-type 129/Ola and 101LL mice to examine the ability of the different recombinant PrP (recPrP) conformers to either seed PrP amyloid plaques, or cause the development of TSE disease in 101LL mice. In contrast to other studies describing inoculation of recPrP fibrils [12, 13, 37, 39, 40, 42, 43, 61, 66], we saw no TSE disease in recipient mice, but did reproduce the seeding of PrP amyloid plaques observed in our previous work following inoculation of brain extract containing in vivo-derived amyloid fibrils.

## Materials and methods

### Transgenic mice

101LL knock-in transgenic mice express the murine PrP gene containing a proline-to-leucine mutation at codon 101. The mice were produced by homologous recombination in embryonic stem cells and have been described previously [44, 48]. 101LL mice are maintained on the same genetic background as control wild-type 129/Ola mice, allowing direct comparison of results between wild-type and mutant mice, without the complication of transgene overexpression, or other effects caused by random integration of the transgene in the murine genome.

### Production and refolding of recPrP isoforms

Cloning and expression of wild-type, murine recPrP has previously been described in detail [33]. An equivalent expression plasmid was created in which the proline at codon 101 was changed to leucine, and both forms of the protein were expressed in Rosetta *Escherichia coli* bacteria. For refolding into monomeric or oligomeric forms, proteins were purified by sequential Ni-IMAC and ion-exchange chromatographies, and the single disulphide bond made by overnight oxidation catalysed by copper ions, as previously published [63]. Copper ions and denaturant were removed by dialysis into 50 mM sodium acetate, pH 5.5, and the final protein snap-frozen prior to use. For refolding into fibrillar isoforms, recPrP was purified by sequential Ni-IMAC and gel filtration after which the single disulphide bond was created using glutathione shuffling; the protein was, then, further purified by reverse-phase HPLC, as per previous reports [23]. Final elution fractions were lyophilised and snap-frozen prior to use. To control for environmental contamination, a saline eluate from the final column was also prepared, which was subjected to the same refolding conditions detailed for each different PrP isoform. This preparation was inoculated into groups of mice to control for possible TSE contamination.

RecPrP in 50 mM sodium acetate, pH 5.5, was shown to possess an α-helical conformation by far-UV circular dichroism (CD) spectroscopy, which produced the expected double minima at 210 and 222 nm. Prior to inoculation, samples were spun briefly to remove aggregates, and passed through a 0.22-µm filter to sterilise. Protein assays indicated concentrations of 1.33 mg/ml for 101L-recPrP, and 0.48 mg/ml for WT-recPrP.

Oligomer formation followed a procedure originally developed by Rezaei et al. [62]. Oligomerisation of recPrP was initiated by buffer exchange of samples at ~2 mg/ml into 50 mM sodium citrate (pH 3.4) [32]. The samples were heated overnight, and an increase in oligomeric PrP forms was confirmed by size exclusion chromatography using a TSKgel G3000SW (Tosoh). Prior to inoculation, samples were spun briefly to remove any larger aggregates that may have formed during storage, and passed through a 0.22-µm filter to sterilise. Protein assays were performed to confirm that protein was recovered following centrifugation and filtration. Concentrations of 0.33 mg/ml for 101L-recPrP, and 0.44 mg/ml for WT-recPrP were obtained.

RecPrP stocks were fibrillised into amyloid by incubation under moderately denaturing conditions with vigorous shaking [23]. Conversion to amyloid was monitored by thioflavin T fluorescence. The presence of fibrils was confirmed by demonstrating that a 16-kDa band was retained following digestion with proteinase K [6]. Fibrillar morphology of the refolded recPrP fibrils (70 μg/ml diluted in 10 mM NaAc buffer) was confirmed by phosphotungstic acid negative staining technique and electron microscopy. Formvar-coated copper grids were placed onto a 50-μl drop of fibril preparation. After 45 s, the grid was removed, touched to a filter paper to remove excess fluid and, then, placed onto a drop of filtered 2 % aqueous phosphotungstic acid for 2 min. Grids were then air-dried before storage and examined using a Jeol 1200EX transmission electron microscope. Since fibrillar PrP samples were composed of large aggregates, no filter sterilisation was performed on these inocula. Instead, samples of inocula were plated on blood agar and incubated both aerobically and anaerobically to confirm lack of bacterial contamination prior to inoculation. Protein concentrations for amyloid preparations were 0.07 mg/ml for the 101L-recPrP and 0.40 mg/ml for the WT-recPrP.

### Inoculation of transgenic mice

Stocks of each 101L-recPrP and WT-recPrP preparation (monomers, oligomers and fibrils) were diluted to 100 µg/ml (α-monomeric), 150 µg/ml (oligomers) and 70 µg/ml (fibrils) for inoculation. Oligomeric preparations contain a small proportion of α-monomeric material (~1:3 ratio monomers:oligomers) and were, therefore, diluted to 150 µg/ml to allow inoculation of equivalent amounts of oligomers to the pure monomer preparations. To control for possible TSE contamination during preparation and refolding, three control buffer eluates from the chromatographic separations were processed through the refolding/misfolding steps required to produce monomers, oligomers and fibrils, and inoculated as contamination controls (monomer control, oligomer control and amyloid fibril control). Nine groups of 48 mice (24 × 101LL and 24 × wild type mice aged approximately 4–10 weeks) were anaesthetised, and each group inoculated with 20 μl of a single recPrP or control preparation. Inoculations were performed manually with a 26-gauge needle into the right cerebral hemisphere (to the right of the midline and centrally between the eye and the ear). Needle guards were used to ensure a consistent depth of penetration (~2 mm). Mice were monitored through recovery from the anaesthetic, and transferred to new cages with fresh bedding, food and water.

For subpassage experiments, 10 % brain homogenates were prepared from the contralateral (non-inoculated) left cerebral hemisphere samples from selected animals that received either recPrP or control inocula as described above. Tissues were selected from two 101LL mice that received 101L-recPrP oligomers, two 101LL mice that received 101L-recPrP or WT-recPrP amyloid fibrils, one 101LL mouse that received control oligomer inoculum, and one 101LL mouse that received control amyloid fibril inoculum. The six inocula were injected intracerebrally (20 µl) into groups of 48 mice (24 × 101LL mice and 24 × wild-type mice at ~4–10 weeks of age) as described above.

At 150 days post-inoculation, a formal clinical monitoring system was started. Animals were scored weekly for clinical signs, indicative of TSE disease, by trained observers according to a previously established TSE clinical scoring system [14]. Observers were also requested to note any other unusual behaviour or neurological signs in these animals. The characteristic signs of clinical TSE infection may include; lethargy, hyperactivity, ataxia, pruritis, gait effect, and aggression depending on the combination of TSE and mouse strain. Non-specific signs in the animal may be observed for a few weeks prior to the development of definite neurological signs which normally occur during the last 2–3 weeks of the incubation period. Animals were scored as 1 (Normal); 2 (possibly affected—evidence of some signs but not necessarily related to TSE); +(definitely affected. Animal shows clinical signs of TSE); g (animal has gait abnormality but not clinical TSE); G (animal has clinical TSE score and gait abnormality). Animals were culled by a schedule 1 method after (a) two consecutive weekly scores of “definitely affected” (+ or G) or (b) after receiving scores of “definitely affected” (+ or G) in two out of three consecutive weeks, or (c) for welfare reasons after consultation with the NACWO. Achieving two “definitely affected” scores increases the confidence that the clinical diagnosis of TSE is correct.

Half the brain was snap-frozen in liquid nitrogen for biochemical analysis or passage (contralateral to injection site), and the remaining half brain (ipsilateral to injection site) was fixed in formol saline and paraffin-embedded for histological processing. When tissue showed autolysis, whole brain was processed for histology. All mouse experiments were approved by the Local Ethical Review committee and performed under Licence from the UK Home Office in accordance with the Animals (Scientific Procedures) Act 1986.

### Vacuolation scoring

Sections (6 µm) were cut from paraffin-embedded mouse brain tissue and stained using haematoxylin and eosin (H&E). Spongiform degeneration was assessed at nine grey-matter regions (medulla, cerebellum, superior colliculus, hypothalamus, thalamus, hippocampus, septum, retrospinal cortex, cingulated and motor cortex) and three regions of white matter (cerebellar white matter, midbrain white matter, and cerebral peduncle). Sections were scored on a scale of 0 (absence) to 5 (severe) for the presence and severity of spongiform degeneration as previously described [8, 20].

### Immunohistochemistry of formol saline immersion-fixed tissue (light microscopy analysis)

Sections (6 µm) were cut from paraffin-embedded mouse brain tissue, autoclaved for 15 min at 121 °C and immersed in 95 % (v/v) formic acid for 10 min prior to overnight incubation with 0.44 g/ml anti-PrP monoclonal antibody (MAb) 6H4 (Prionics) at room temperature. Secondary anti-mouse biotinylated antibody (Jackson Immuno Research Laboratories, UK) was added at 2.5 g/ml and incubated for 1 h at room temperature. Immunolabelling was performed using the ABC Elite kit (Vector Laboratories), and the signal was visualised by a reaction with hydrogen peroxidase-activated diaminobenzidine. Sections were blinded and examined for PrP deposition without knowledge of genotype or inoculum.

### Immunohistochemistry of paraformaldehyde/glutaraldehyde perfusion-fixed tissue (light and electron microscopic analysis)

Brain tissue from two 101LL mice inoculated with 101L amyloid fibrils, two 101LL mice inoculated with wild-type amyloid fibrils, and two wild-type mice inoculated with 101L amyloid fibrils were perfusion-fixed in 4 % paraformaldehyde/0.1 % glutaraldehyde at cull >600 days following intracerebral challenge. Alternate fixed serial coronal brain slices (1 mm) were embedded in paraffin wax or were further trimmed into 1-mm^3^ blocks, post fixed in osmium tetroxide and embedded in araldite resin.

#### Light microscopy (wax)

Wax-embedded blocks were cut and stained with H&E or were labelled using a light microscopic immunohistochemical procedure as described previously [22]. PrP MAbs SAF84 (Bertin Pharma, Montigny le Bretonneux, France) and 1C5 (Y.S. Kim, Hallym University, Republic of Korea), and polyclonal antibodies 1A8 [18] and R523.7 (J. Langeveld, ID–Lelystad, Netherlands) were applied overnight at 27 °C, at dilutions of 1:2000, 1: 1000, 1:1000 and 1:12,000, respectively. Polyclonal anti-glial fibrillary acidic protein (GFAP) and anti-ubiquitin antibodies (both Dako, Ely, Cambridgeshire, UK) were also applied at dilutions of 1:8000 or 1:500 (respectively).

#### Light microscopy (resin )

As described previously [46], the avidin–biotin complex immunohistochemical staining method was applied to the etched and pre-treated sections using SAF84 at a dilution of 1:400 and 1A8 at a dilution of 1:2000. Mice infected with 87 V mouse scrapie were used as positive controls due to the PrP amyloid plaque pathology.

#### Ultrastructural microscopy

At least three blocks of corpus callosum or hippocampus containing immunolabelled plaques were identified in each of the two 101LL mice inoculated with 101L-recPrP amyloid fibrils. Blocks containing corpus callosum and hippocampus were also taken for analysis from both 87 V controls and from the wild-type mouse inoculated with 101L-recPrP amyloid fibrils. Multiple sections (65 nm) from each block were stained using uranyl acetate and lead citrate, or immunolabelled using 1A8 as described previously [46]. A pre-immune serum was applied to each section as a control. Grids were examined using a Jeol 1200EX transmission electron microscope.

### Detection of amyloid plaques by thioflavin-S fluorescence

Amyloid deposits in tissue sections were observed using thioflavin-S. Briefly, following haematoxylin staining, sections (6 µm) were immersed in 1 % (w/v) thioflavin-S (Sigma, UK) solution as previously described [54]. Sections were mounted and viewed under a fluorescence microscope.

### Detection of PK-resistant PrP by histoblotting

Sections (6 µm) were cut from paraffin blocks onto strips of nitrocellulose membrane. These were processed as described previously [65], and digested with PK (20 µg/ml) overnight at 55 °C. Blots were probed with anti-PrP Mab BH1 (1/3000) [45] and plaques visualised with goat anti-mouse alkaline phosphatase secondary (1/5000) and NBT/BCIP tablets (Sigma).

### PCR genotyping of mouse tail DNA

All mice were analysed by PCR postmortem to confirm PrP genotype. Mouse tail DNA was extracted and genotype verified by PCR as previously described [44].

## Results

### Inoculation of α-monomeric, oligomeric and fibrillar recPrP preparations does not induce TSE disease in mice

**Fig. 1:**
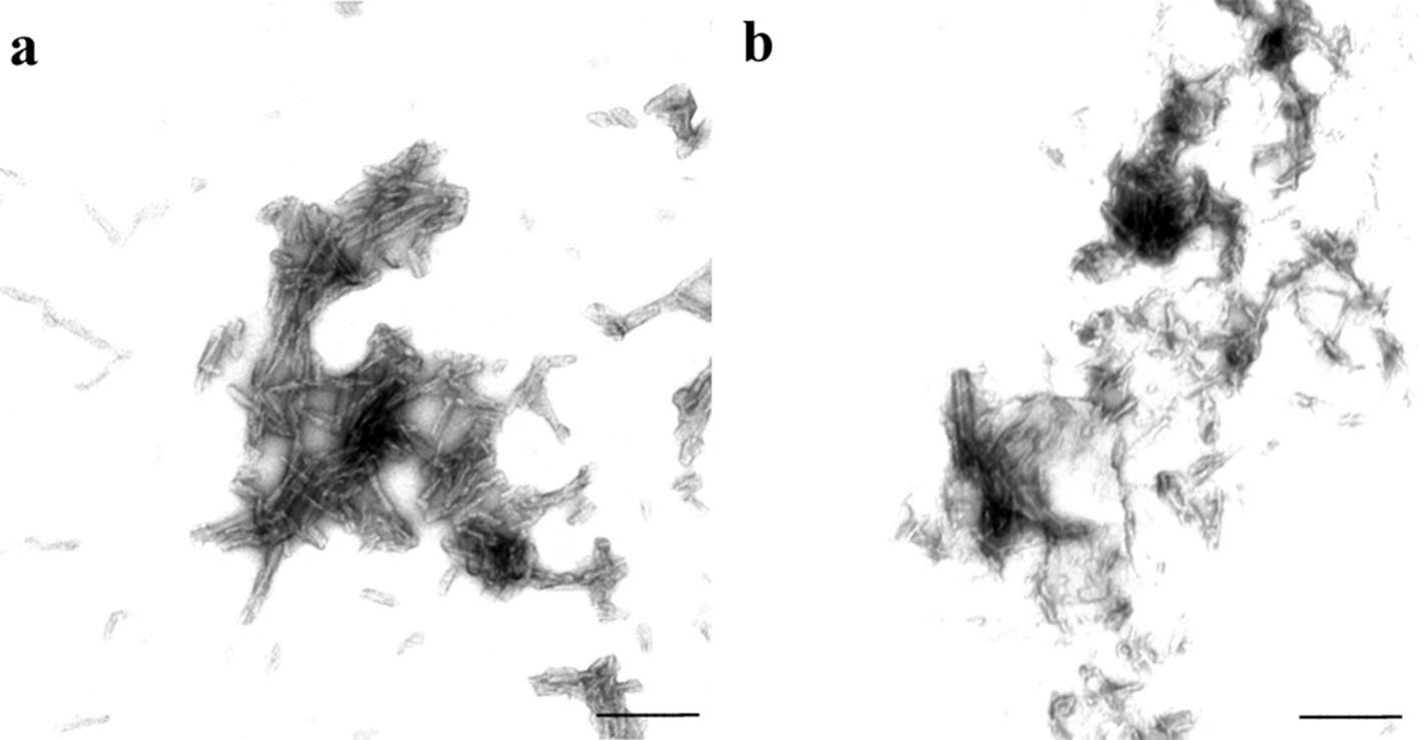
RecPrP refolded into amyloid fibrils was examined for fibrillar morphology by EM. Phosphotungstic acid-stained fibrils from 101L-recPrP (**a**) and WT-recPrP (**b**). *Scale bars* 200 nm

Purified PrP fibrils were produced from recPrP to examine whether PrP aggregates alone could seed PrP amyloid formation or induce TSE disease in 101LL mice. WT-recPrP and 101L-recPrP proteins were expressed, purified and refolded into a native-like α-helical, monomeric isoform, as well as isoforms possessing oligomeric and amyloid fibrillar morphologies. Tertiary/quaternary structures of the proteins were confirmed by far-UV circular dichroism (CD) spectroscopy and size exclusion chromatography for monomers and oligomers. Fibril preparations were analysed by both limited protease digestion and electron microscopy to confirm fibrillar structure (Fig. 1). Each refolded recPrP preparation, plus control eluates from the chromatographic purification steps (9 inocula in total), were inoculated into groups of 24 101LL and 24 wild-type mice (Table 1). Experiments were terminated at approximately 700 days post-inoculation. Of the 432 mice inoculated, 54 mice died due to intercurrent illness with no tissue harvested. The remaining 378 animals were culled for welfare reasons (between 126 and 702 days post-inoculation), except for three 101LL mice (one each inoculated with WT-recPrP oligomer, 101L-recPrP oligomer and oligomer saline control) that were culled showing possible clinical signs of TSE disease (605, 619 and 637 days old). No other adverse behavioural or neurological phenotypes were observed in any of the mice. Brain tissue was removed from all 378 mice and subjected to histopathological analysis for TSE disease pathology. None of the 378 mice examined, including the three mice culled showing possible clinical signs of disease, showed any TSE-associated spongiform degeneration in the brain regardless of the sequence or conformer of recPrP inoculated, or genotype of recipient mouse.

**Table 1:** Inoculation of WT-recPrP and 101L-recPrP isoforms

Inoculum	Recipient	Survival (±SEM)^a^	Spongiform degeneration	PrP amyloid plaques
WT monomer	101LL	586 ± 26	0/21	0/21
	129/Ola Wt	518 ± 24	0/21	0/21
101L Monomer	101LL	608 ± 20	0/22	0/22
	129/Ola Wt	603 ± 31	0/22	0/22
Monomer control	101LL	558 ± 30	0/19	0/19
	129/Ola Wt	653 ± 19	0/21	0/21
WT oligomer	101LL	509 ± 25	0/19	0/19
	129/Ola Wt	526 ± 29	0/22	0/22
101L oligomer	101LL	550 ± 18	0/21	0/21
	129/Ola Wt	587 ± 20	0/22	0/22
Oligomer control	101LL	543 ± 15	0/24	0/24
	129/Ola Wt	559 ± 23	0/23	0/23
WT amyloid fibril	**101LL**	**508 ± 19**	**0/21**	**10/21**
	129/Ola Wt	605 ± 17	0/20	0/20
101L amyloid fibril	**101LL**	**519 ± 27**	**0/19**	**14/19**
	129/Ola Wt	560 ± 30	0/18	0/18
Amyloid fibril control	101LL	537 ± 25	0/19	0/19
	129/Ola Wt	628 ± 15	0/24	0/24

Bold highlights transmissions which resulted in amyloid plaque deposition in 101LL mice

^a^All primary experiments terminated ~700 dpi. Survival termed as time from inoculation to cull

## 101LL mice inoculated with recPrP amyloid fibrils form PrP amyloid plaques in brain tissue

**Fig. 2:**
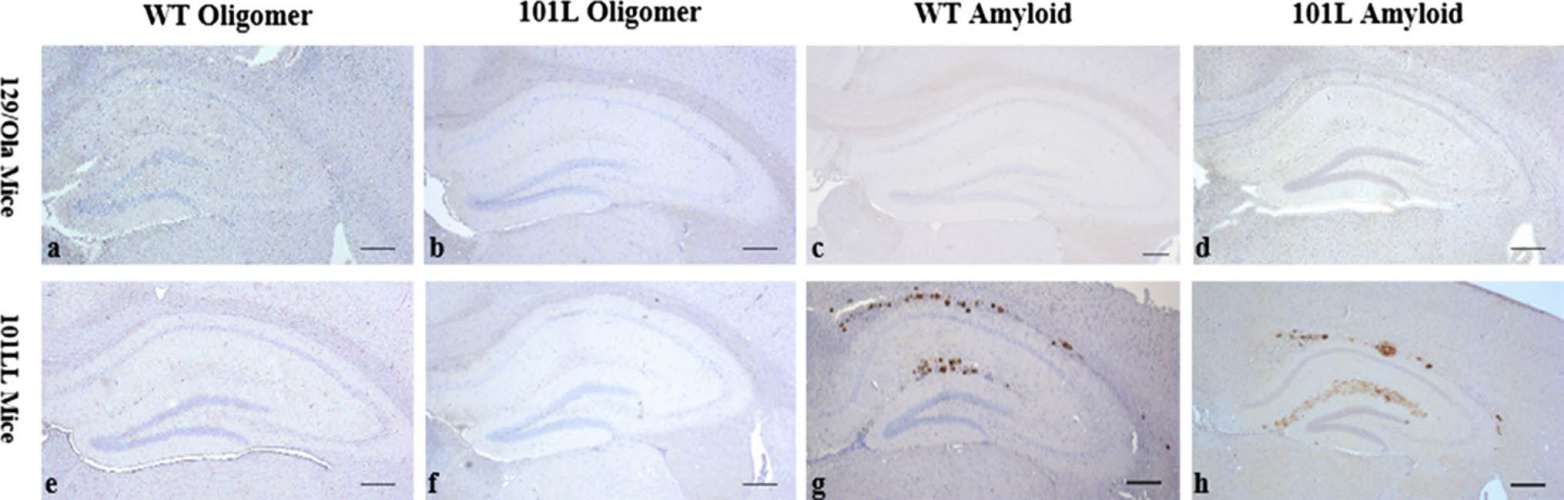
Immunohistochemical analysis of brain sections from 101LL or wild-type mice inoculated with oligomeric (**a**, **b**, **e**, **f**) or fibrillar (**c**, **d**, **g**, **h**) refolded recPrP preparations. Abundant plaque deposition was observed only in 101LL mice that received fibrillar preparations (**g**, **h**), and was most prominent in the corpus callosum and hippocampus. Sections stained with anti-PrP Mab 6H4. *Scale bar* 200 µm

**Fig. 3:**
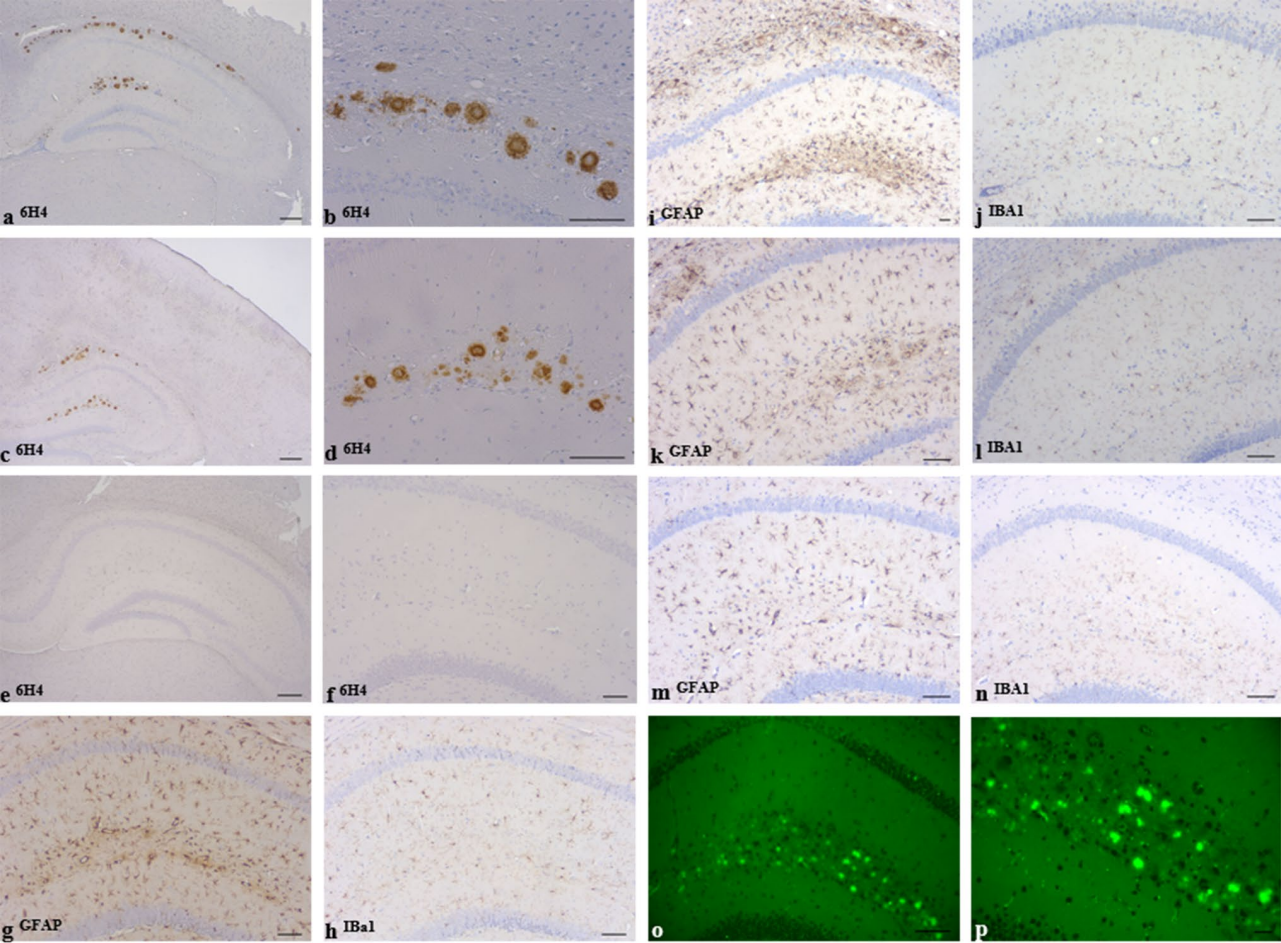
Immunohistochemical analysis of PrP deposition and glial activation in 101LL mice inoculated with WT-recPrP fibrils (**a**, **b**, **i**, **j**), α-monomeric recPrP (**e**, **f**, **m**, **n**), or following subpassage from 101L-recPrP fibril-inoculated 101LL mice with plaque deposition (**c**, **d**, **k**, **l**). Similar patterns of PrP plaque seeding were observed in 101LL mice on primary inoculation (**a**, **b**) and subpassage (**c**, **d**) of WT-recPrP fibrils. Thioflavin-s fluorescence of plaques in 101L-recPrP fibril-inoculated 101LL mice (**o**, **p**). Similar amounts and type of glial reactivity were seen in age-matched, control un-inoculated 101LL mice (**g**, **h**) and inoculated 101LL mice (**i**–**n**). Stained with anti-PrP Mab 6H4 (**a**–**f**); GFAP (astrocytes) (**g**, **i**, **k** and **m**); Iba I (microglia) (**h**, **j**, **l** and **n**). *Scale bars* 100 μm (**a**, **c**, **e**, **o**, **p**); 50 μm (**b**, **d**, **f**–**n**)

Brain sections from all 378 mice were analysed for PrP deposition by immunostaining with anti-PrP Mab 6H4. All mice inoculated with α-monomeric recPrP, oligomeric recPrP or saline control inocula were negative for any forms of PrP deposition in brain tissue by immunohistochemistry (IHC) and showed no thioflavin-s fluorescence regardless of sequence of recPrP or recipient mouse genotype (Figs. 2, 3). Hence, preparations containing only monomeric and oligomeric isoforms, generated under our experimental conditions, were unable to elicit neurological disease, spongiform degeneration, or induce PrP aggregation in wild-type or 101LL mice. However, some 101LL mice inoculated with either WT-recPrP or 101L-recPrP amyloid fibrils showed large thioflavin-s fluorescent PrP amyloid plaques that were absent in wild-type mice that received the same inocula (Figs. 2, 3). Large multicentric plaques were observed in 10/21 101LL mice that received WT-recPrP amyloid fibrils, and in 14/19 101LL mice that received 101L-recPrP fibrils (Table 1). Plaques were present mainly in the corpus callosum and vicinity, as well as in some areas of the stratum lacunosum and stratum moleculare of the hippocampus. In a few animals, plaques were also visible in the subventricular area. Plaque morphology was variable, and composed of unicentric, stellate and multicentric deposits. Bilateral distribution of plaques (in the corpus callosum, hippocampus and striatum) was observed in animals for which whole brain slices were available (Figure S1). Analysis by histoblot showed plaques to be formed of proteinase K-resistant PrP (Figure S2). Gliosis and glial cell activation was similar to that observed in aged matched un-inoculated control 101LL mice (Fig. 3). No obvious difference in plaque morphology or distribution was observed in recipient 101LL mice following challenge with either WT-recPrP or 101L-recPrP preparation. Overall, despite the absence of TSE disease, PrP amyloid plaques were induced in 101LL mice following inoculation of recPrP fibrils.

### Plaques formed in vivo by recPrP fibril seeding do not cause TSE disease on subpassage

Previous experiments by other researchers have demonstrated the development and serial transmission of TSE disease in hamsters and mice following inoculation of “synthetic prions” created from refolded recPrP fibrils [12, 13, 37, 39, 40, 42, 61]. We observed no clinical signs of TSE or spongiform degeneration following primary inoculation of mice with the different recPrP conformers described here, yet PrP amyloid plaques were observed in some 101LL mice. Subpassage of tissues from 101LL mice that received refolded oligomeric and amyloid recPrP isoforms was, therefore, performed to determine whether these tissues harboured low levels of TSE infection and could transmit TSE disease. We prepared inocula from brain tissue of two 101LL mice that had received 101L-recPrP oligomers (inocula 1 and 2) and two 101LL mice that had received either 101L (inoculum 3) or WT-recPrP amyloid fibrils (inoculum 4) (Table 2). The two mice that received recPrP amyloid fibrils were culled at 520 (inoculum 3) and 516 (inoculum 4) days post-inoculation, and both had PrP amyloid plaques identified in the brain by immunostaining. Two corresponding buffer control-inoculated mice (inoculum 5 produced under amyloid refolding conditions, inoculum 6 under oligomer refolding conditions) were also selected as negative controls (Table 2). The six inocula were injected intracerebrally into groups of 24 × 101LL mice and 24 x wild-type mice, and animals were monitored for clinical signs of TSE disease. Experiments were terminated approximately 500 days post-inoculation. Of the 288 mice inoculated, 16 died due to intercurrent illness, and no tissues were harvested. Neurological signs suggestive of TSE disease were observed in 8 of the remaining 272 mice (4 from oligomer subpassage and 4 from amyloid subpassage; 442–619 days old).

**Table 2:** Subpasage of brain from recPrP-inoculated 101LL mice

Inoculum	Source (mouse line/inocula)	Recipient	Spongiform degeneration	PrP amyloid plaques
1	101LL/101L oligomer	101LL	0/24	0/24
		129/Ola WT	0/20	0/20
2	101LL/101L oligomer	101LL	0/23	0/23
		129/Ola WT	0/22	0/22
**3**	**101LL/101L amyloid fibril**	**101LL**	**0/24**	**18/24**
		129/Ola WT	0/22	0/22
**4**	**101LL/WT amyloid fibril**	**101LL**	**0/23**	**17/23**
		129/Ola WT	0/23	0/23
5	101LL/Amyloid fibril control	101LL	0/23	0/23
		129/Ola WT	0/22	0/22
6	101LL/Oligomer control	101LL	0/24	0/24
		129/Ola WT	0/22	0/22

Bold highlights transmissions which resulted in amyloid plaque deposition in 101LL mice

All subpassage experiments terminated ~500 dpi

None of the 272 mice available for analysis showed any TSE-associated spongiform degeneration in the brain. Following immunostaining of brain sections, PrP amyloid plaques were observed in 18/24 101LL mice that received inoculum 3, and 17/23 101LL mice that received inoculum 4 (Table 2; Fig. 3). No PrP plaques were observed in any wild-type mice that had been inoculated with inocula 3 or 4, or any mice (101LL and wild-type) inoculated with inocula 1, 2, 5 or 6. Two of the mice culled showing possible clinical signs of TSE (culled at 468 days post inoculation) were subsequently shown to have PrP amyloid plaques in the brain. However, a further 33 mice from the subpassage of the recPrP fibrils (29 of which survived >468 days) also showed seeding of PrP amyloid plaques, but had no associated clinical signs of TSE disease. There was also no significant difference between the number of possible clinical cases in all 101LL vs wild-type groups (Students *t* Test, *p* = 0.24) and also no correlation between possible clinical signs and plaques in the 101LL mice (Chi-squared test, *p* = 0.24). These neurological signs were probably due to intercurrent illness or age-related issues. The inoculation of 101LL brain containing recPrP fibril seeded plaques was, therefore, unable to cause TSE disease in recipient mice.

### Ultrastructural pathology following seeding with recPrP fibrils is similar to that seen following seeding with P102L and P101L PrP brain-derived fibrils

Electron microscopy (EM) was used to examine the brain tissue ultrastructure in four 101LL mice that received WT-recPrP or 101L-recPrP amyloid fibril inocula, and two wild-type mice that received 101L-recPrP fibril inocula. Plaques were identified in three of the four 101LL mice, but not in wild-type mice. All plaques and other accumulations of PrP were located mainly in the corpus callosum and stratum lacunosum of the hippocampus.

**Fig. 4:**
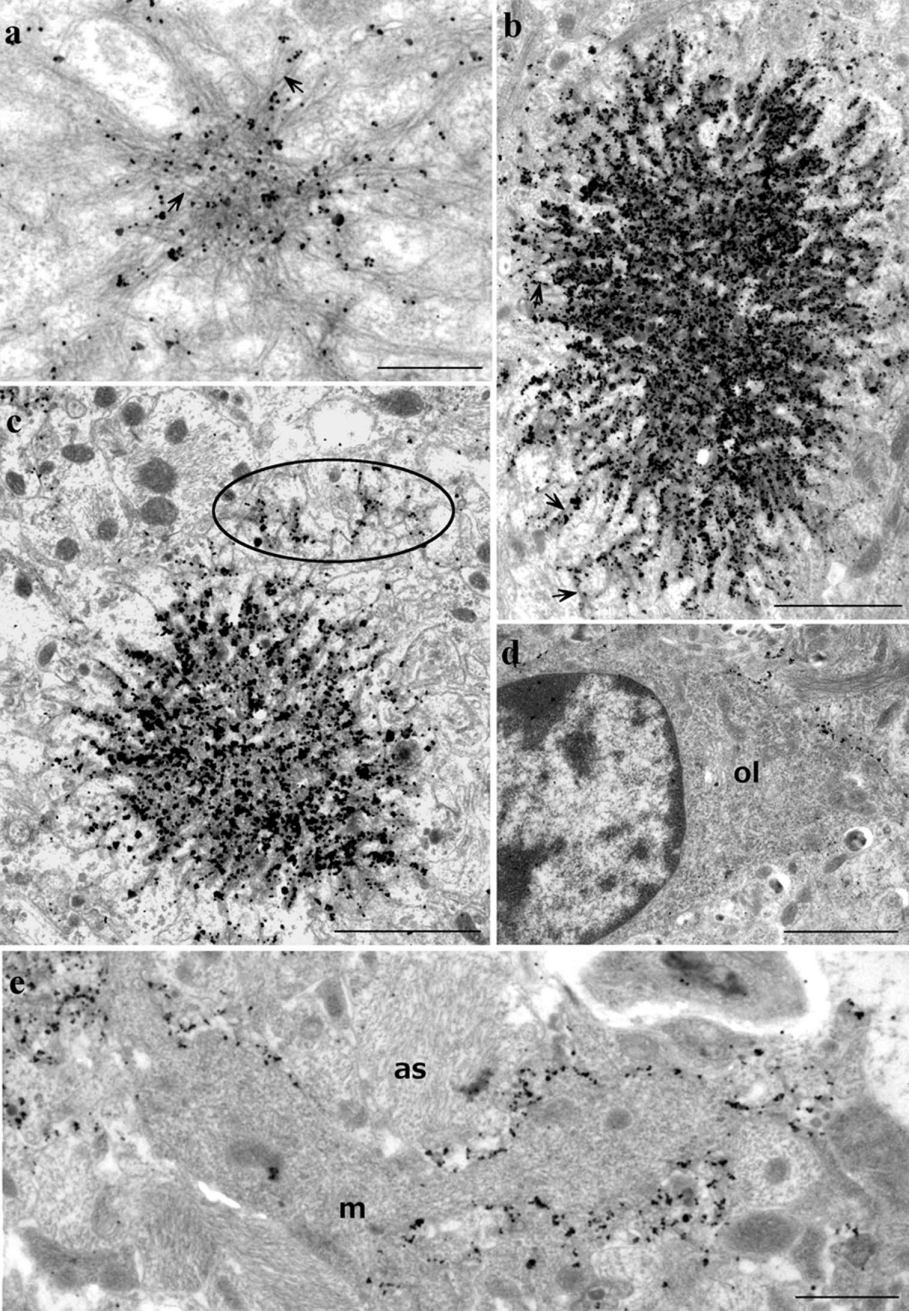
Brain tissue from 101LL mice inoculated with WT-recPrP or 101L-recPrP fibrils was processed for immunogold EM analysis. Immunogold labelling of PrP showed small and large stellate plaques (**a**, **b**) with intense labelling of densely packed cores (which was not evident from light microscopy studies). *Arrows* indicate individual amyloid fibrils. Membrane immunogold labelling was evident in the absence of amyloid fibrils adjacent to plaques (**c** *circled*) and also occurred in neuropil unconnected to plaque deposits (**d**, **e**) where it was mainly located to (**d**) oligodendrocyte membranes (ol), (**e**) astrocytes (as), and occasionally to microglia (**m**). *Scale bars* 500 nm (**a**), 2 µm (**b**–**d**), 1 µm (**e**)

Two types of plaques were identified by EM following both WT-recPrP and 101L-recPrP amyloid fibril inoculation: large multicentric plaques consisting of a dense central core of interweaving bundles of amyloid fibrils with smaller amyloid cores adjacent to the main plaque. Also, smaller, stellate plaques consisting of radially arranged bundles of amyloid fibrils were present (Fig. 4). Surrounding mature plaques were many dystrophic neurites containing large numbers of lysosomes and other electron-dense organelles and degenerate myelinated axons. Plaques were surrounded by reactive astrocytes and microglia. Also, granular PrP labelling seen by light microscopy corresponded to diffuse areas of loosely arranged amyloid fibrils and non-amyloid cell membrane associated PrP accumulations that were detected only by immunogold electron microscopy.

Immunogold labelling confirmed localisation of aggregated PrP to amyloid fibrils within plaques, but labelling for aggregated PrP extended beyond the amyloid fibrils and was present on membranes of cellular processes at the extreme periphery of the plaque where no visible amyloid fibrils were present (Fig. 4). Where plaques were located in the corpus callosum, these labelled cells and cell processes belonged to astrocytes or oligodendroglia (Fig. 4), suggesting that these cells are the continuing source of fibrillar PrP found in mature white matter plaques of the corpus callosum. Dendritic processes were also labelled around plaques located in the hippocampal grey matter.

Numerous foci of glial membrane PrP labelling were identified in the absence of plaques. Many of these labelled membranes were abnormal: processes were highly irregular, and many small diameter profiles were present, and polyp-like protrusion of glial membranes extended from processes. Aggregated PrP immunolabelling was particularly associated with these small irregular membrane microfolds or polyps. In rare instances, accumulation of aggregated PrP was seen on membranes lacking any other visible membrane changes that could be confirmed by EM.

As described previously, and in contrast with classical murine scrapie, no intra-lysosomal PrP accumulation was evident in any of the mice with plaques, and abnormal membrane pathology associated with infectious TSE disease (tubulovesicular bodies, spiral membrane invaginations, increased coated pits) was also absent. Overall, the ultrastructural pathology was more prominent, but similar to that previously described in 101LL mice with plaques seeded following inoculation of P102L GSS brain-derived fibrils [30].

## Discussion

Previous work in our laboratory has demonstrated that misfolding and aggregation of PrP can occur in the absence of TSE agent replication and infectious TSE disease [30, 55, 56]. These experiments involved inoculation of brain material derived from cases of neurological disease (GSS patients and sick GSS22 mice overexpressing 101L-PrP), which contained other tissue components in addition to amyloid protein seeds. Using refolded WT-recPrP and 101L-recPrP fibrils as inocula, we have now demonstrated seeding of PrP amyloid plaques in 101LL mice (but not wild type mice), proving that the misfolded protein seed alone can initiate plaque formation. No such seeding was produced following inoculation of recPrP oligomers or control α-helical PrP isoforms, indicating that amyloid fibrils, or specific, smaller protofibrils are required to initiate seeding. Aggregation of 101L-PrP in the host was also seeded using both WT-recPrP and 101L-recPrP fibrils as inoculum, indicating that it is the structure of the inoculated seed, not the amino acid sequence, that is important in initiating aggregation. Hence, both the macromolecular structure of PrP in the inoculum and the expression of mutant 101L-PrP in the host are required for efficient amyloid plaque formation. Whether seeding would have been observed in wild-type mice if lifespan had been extended is unknown, but possible. The 101L mutation in murine PrP may alter the kinetics of aggregation, or cellular processes involved in the clearance of protein aggregates, resulting in an acceleration of the process. These issues will be examined in future studies of this model.

The creation of “synthetic prions” has been described previously by others following inoculation of amyloid fibrils derived from refolded recPrP sources [12, 13, 37, 39, 40, 42, 43, 61, 66]. In contrast to our study, these preparations were shown to cause a neurological disease/TSE on primary inoculation or subpassage in mice. One recent study has also shown development of neurological disease following inoculation of recPrP oligomers rather than fibrils, but only following refolding in the presence of RNA molecules extracted from purified 263K scrapie fibrils [66]. It is difficult to draw general conclusions from these experiments as in each case, the conditions used to refold recPrP prior to inoculation were different, with varying concentrations of denaturant, different buffering, and variations in speed of agitation [12, 37, 39, 42, 61, 66]. Inoculations were performed in different mouse lines [12, 13, 37, 61], and hamsters [39, 40, 42, 43, 66], using amounts of recPrP ranging from 0.2 µg [66] to 30 µg [12]. Following recPrP fibril inoculation, some studies [39, 40, 42, 61] did show evidence of PrP aggregation in brain in the absence of TSE disease, agreeing with the data presented here. However, in contrast to our study where only plaque formation was observed on subpassage, neurological disease/TSE was observed in mice following subpassage of these other models [39–41, 61]. A recent study which examined 19,468 unique refolding conditions and assayed by infection in cell culture concluded that none of the conditions reproducibly created high-titre infectious synthetic prions, and that creation of synthetic infectivity is a rare event which cannot be efficiently reproduced in vitro [64].

EM analysis of subpassage tissue from 101LL mice with seeded plaques confirmed that amyloid plaques (including multicentric plaques) seeded by recPrP shared the same origin on cell membranes, the same growth by conversion of native cell-membrane PrP^C^ on cellular processes and seed dispersal through the interstitial spaces as those previously described in 101LL mice inoculated with atypical GSS [30]. Similarly, 101LL mice showed no abnormal membrane pathology and intra-lysosomal PrP labelling, both associated with TSE disease [30, 31]. The generation of abnormal PrP predominantly by oligodendroglial cells is also atypical of that of classical, naturally occurring TSEs, where most abnormal PrP is formed on the surface of neuronal dendrites. These data suggest that recPrP fibrils and GSS inocula seed plaque formation by a common mechanism, and that plaques in GSS-inoculated 101LL mice were formed from amyloid seeds in the atypical GSS inoculum, and not from other components of the inoculum. Although no TSE disease was observed in atypical GSS-inoculated 101LL mice, it should be noted that these isolates do appear to be transmissible in different animal models [58]. Interestingly, the lack of abnormal membrane pathology and intra-lysosomal PrP staining was also observed following pathological analysis of hamsters with SSLOW (Synthetic Strain Leading to Overweight) initiated with refolded recPrP fibrils [39–41]. In addition, the localisation of plaques in SSLOW hamsters was also distinct from most TSE disease models, being found predominantly in the glial limitans and microfolded astrocytic processes. The pattern of deposition of amyloid plaques in SSLOW hamsters and recPrP fibril-inoculated 101LL mice may, therefore, be due to distribution of amyloid seeds through the extracellular space following high-volume intracerebral inoculation. Indeed, distribution of plaques in 101LL mice mimics the distribution of India ink observed by others when inoculated into murine brain [71]. Other studies have shown that following injection into the brain, inocula are drained via perivascular pathways or leak into the CSF and enter the blood [72]. Once in the blood, seeds may re-enter the brain through the circumventricular organs (CVOs) due to the incomplete blood brain barrier at this site [67, 68]. The variation in pattern and intensity of plaque deposition between different recPrP preparations could, therefore, be determined by the aggregate size (due to variations in the refolding conditions) and ability to spread via the CSF, interstitial fluid or blood giving rise to deposition in further areas of the brain via the CVOs.

Our observations of plaque formation in 101LL mice in the absence of TSE disease show similarities to work performed, by others, in transgenic mice expressing human amyloid precursor protein (huAPP). Plaques can be induced in transgenic mice expressing wild-type huAPP and huAPP with familial AD mutations following inoculation with AD brain homogenate or aged huAPP Tg brain homogenate [47, 49, 71]. Initial attempts to induce seeding in AD Tgs using recombinant Aβ were unsuccessful [47]. However, recent studies have demonstrated accelerated seeding in APP23 mice expressing a GFAP-luc transgene using high levels (7.5 μg) of aggregated recombinant Aβ 1–40 monomers or mutant Aβ 1–40 dimers (AβS26C)_2_ [69, 70]. Seeding of Aβ plaques may, therefore, occur via a mechanism similar to seeding of non-pathogenic PrP amyloid plaques in 101LL mice. Recent studies examining archival material from cases of iatrogenic CJD (iCJD) have also shown the probable seeding of Aβ plaques in humans following the administration of human pituitary-derived growth hormone [29] or the implantation of dura matter grafts [21, 35]. Whilst these materials appear to be the source of Aβ seeds, and responsible for the formation of Aβ plaques in brains of the recipients, other signs of AD pathology were not present. In particular, in the detailed pathological analysis of two cases of iCJD that developed following dura matter grafting described by Kovacs et al. [35], the authors state that Aβ seeding is unable to reproduce the full clinicopathological phenotype of AD.

We have shown that PrP amyloid can be seeded in the brains of 101LL mice in the absence of neurological signs of TSE disease, spongiform degeneration of the brain and TSE infectious agent replication. Importantly, we have established that such seeding can occur not only following inoculation of brain extracts from human (atypical P102L GSS) and murine (GSS-22) sources, but also from non-brain-derived recPrP fibrils. Our data provide no evidence for the generation of “infectious prions” following inoculation and subpassage of synthetic PrP amyloid fibrils; instead, we observe the induction and maintenance of a seeded proteinopathy [30, 55, 56]. The development of neurological disease in some synthetic prion models may, therefore, depend on the severity and distribution of PrP aggregates in the brain, due to the concentration, volume and distribution of seeds inoculated. Indeed, GSS22 Tg mice, which overexpress 101L PrP ~12-fold, develop neurological signs, spongiform degeneration and PrP amyloid plaques, but do not transmit TSE disease on subpassage [53]. Material from the brains of these mice can seed plaques in low-expressing (~2-fold) 101L Tg mice and knock-in 101LL mice [53, 55], indicating that high levels of plaque deposition may, indeed, lead to neurological signs, but not to an infectious TSE disease. These data have led us to hypothesise that there are three potential pathways associated with protein aggregation in the CNS; (a) resulting in an infectious TSE disease and replication of the TSE infectious agent; (b) the induction of a proteinopathy in which large accumulations of amyloid lead to toxic effects in the brain, but are not infectious; (c) the seeding of protein accumulations in the brain that are neither infectious nor toxic (as determined by absence of neurological signs and spongiform degeneration of the brain). On this basis, protein misfolding and aggregation in the brain does not invariably lead to replication of an “infectious prion”, even when the protein in question is PrP. This may explain why some prion diseases (particularly in humans) appear to be non-transmissible, such as atypical P102L GSS, A117V GSS and P105L GSS [34, 56] (P105L GSS: Barron, unpublished data). However, transmission of atypical P102L GSS has recently been demonstrated in bank voles [58] indicating that transmission of such cases may be possible in the right host species. Overall, our results and those of others where plaque formation is seeded by inoculation of pre-formed protein fibrils (or propagons [16]) are consistent with a seeded proteinopathy, in which native proteins can be converted into misfolded aggregates, but does not result in a contagious, naturally transmissible neurodegenerative disease. Hence, it may be preferable to refer to the formation of misfolded protein aggregates in APP transgenic mice and 101LL mice as “seeded proteinopathy”, rather than “prion-like transmission”, to distinguish this mechanism from a truly infectious TSE disease which is transmissible from one individual to another.

## Electronic supplementary material

Below is the link to the electronic supplementary material.

Supplementary material 1 (PDF 3357 kb)
