# Supplementary material for: PrP aggregation can be seeded by pre-formed recombinant PrP amyloid fibrils without the replication of infectious prions
Source: Acta Neuropathol. 2016 Jul 4;132(4):611–24. doi: 10.1007/s00401-016-1594-5 (PMC5023723; doi:10.1007/s00401-016-1594-5)
Supplement: Supplementary file 1 — Supplementary material 1 (PDF 3357 kb) [file 401_2016_1594_MOESM1_ESM.pdf]

Supplementary Information

Acta Neuropathologica 2016

PrP aggregation can be seeded by pre-formed recombinant PrP amyloid fibrils without the replication of infectious prions

Rona M Barron[1*], Declan King[1], Martin Jeffrey[2], Gillian McGovern[2], Sonya Agarwal[1], Andrew C Gill[1] and Pedro Piccardo[1]

[1]The Roslin Institute and R(D)SVS, University of Edinburgh, Easter Bush, Midlothian, EH25 9RG.

[2]Animal and Plant Health Agency, Pentlands Science Park, Midlothian.

Files:

Supplemental Methods and Results

Supplementary Figure 1

Supplementary Figure 2

Data relating to the purification and characterisation of recombinant PrP isoforms used in Barron et al.

This work used 3 different refolding methods to produce recPrP in a physiological α-helical conformation, a β-oligomeric conformation or an amyloid fibril form. This, in turn, necessitated following 3 different purification protocols that were prevalent in the literature at the time when these preparations were made. These protocols are described, in detail, in the following section. Each protocol was applied to the purification of both wildtype (WT-recPrP) and 101L mouse PrP (101L-recPrP). The subsequent data relates to the actual purifications used to generate the protein preparations that were inoculated into mice. In each case, we present chromatographic traces of steps carried out on AKTA FPLC and HPLC instruments, where protein elution was monitored by UV absorbance at 280 nm. Where appropriate, we also monitored conductivity. The percentage of the "B" buffer is given to indicate when elution starts. In almost all cases, SDS-PAGE gels are presented tracking the elution of the protein, apart from 1 purification were gels are not available. On the gels, samples highlighted with an asterisk were those taken forward to the next stage of purification or for refolding. In all cases, MoPrP migrates to ~25 kDa, consistent with its calculated molecular weight of 23 kDa.

PrP expression & lysis

For all preparations Escherichia coli expression bacteria (Rosetta strain) were grown (37 °C, shaking at 225 rpm) in 400 ml volumes of Terrific Broth (Sigma) containing ampicillin, to an OD_{600} of between 0.6-1.0. PrP expression was induced by addition of isopropyl thiogalactoside (IPTG) to a final concentration of 1 mM. Cells were left for at least 4 hours and up to overnight (~16 hours) and were harvested by centrifugation (15 min, 12k rpm, SLA1500 rotor). Cells were resuspended in 10 ml of lysis buffer (50 mM Tris-HCl pH 8, 100 mM sodium chloride, 1 mM ethylenediaminetetraacetic acid (EDTA)) per gram of cell pellet. Lysozyme was added to a final concentration of 20 µg/ml final and the suspension was incubated at 4 °C for 30 min. Sodium deoxycholate (1 mg/ml final) and DNase (5 µg/ml final) were added and 1 M $MgSO_4$ was added to a final concentration of 1 mM. After 30 minutes incubation at room temperature, the inclusion bodies were isolated by centrifugation (15 min, 12k rpm, SLA1500 rotor).

Purification of PrP for refolding to α-monomeric protein

This purification regime was standard in our laboratory to produce protein that was natively folded. Inclusion bodies were solubilised in 10 ml of IMAC buffer A (100 mM sodium phosphate, 10 mM Tris, 8 M urea, 10 mM β-mercaptoethanol, pH 8.0) per gram of pellet, at room temperature for 1-2 hours using constant stirring. Insoluble material was removed by centrifugation (15 min, 12k rpm, SLA1500 rotor) and the supernatant was applied to a home-poured Nickel NTA affinity column (Qiagen). Bound material was eluted by a step elution to IMAC buffer B (100 mM sodium phosphate, 10 mM Tris, 8 M urea, 10 mM 2-mercaptoethanol, pH 4.5). PrP-containing fractions, as assayed by SDS-PAGE, were pooled and diluted 1:1 with IE A buffer (8 M urea, 50 mM HEPES, pH 8.0). This solution was applied to a home-poured cation exchange column (SP-sepharose, Pharmacia). Bound proteins were eluted by a gradient elution to 50 % IE B buffer (8 M urea, 50 mM HEPES, pH 8.0 containing 1 M NaCl). PrP containing fractions, as assayed by SDS-PAGE, were pooled and the protein was diluted to a concentration of ~0.1 mg/ml by the addition of 8 M urea. 5-fold molar excess of copper ions ($CuCl_2$) were added and the solution was stirred overnight to allow copper-catalysed formation of the disulphide bond. The oxidised protein was dialysed extensively against 50 mM sodium acetate, pH 5.5 (6 changes of buffer, the first two of which contained 1 mM EDTA to remove copper ions) and finally the protein was concentrated by use of an Amicon filtration cell. The final concentrations of WT-recPrP and 101L-recPrP were 0.55 and 1.35 mg/ml respectively. The proteins contained predominately α-helical structure as confirmed by circular dichroism spectropolarimetry. Prior to inoculation the proteins were filtered (0.22 µm) and the final concentrations of protein were 0.48 and 1.33 mg/ml for WT-recPrP and 101L-recPrP protein respectively.

Purification of PrP for refolding to β-oligomeric protein

This procedure follows that published by Rezaei et al (Rezaei H, Eghiaian F, Perez J, Doublet N, Choiset Y, Haertle T, Grosclaude J (2005) Sequential generation of two structurally distinct ovine prion protein soluble oligomers displaying different biochemical reactivities. J Mol Biol 347: 665-679 Doi 10.1016/j.jmb.2004.01.043). Inclusion bodies were re-suspended in 10 ml/g IMAC buffer A (9 M urea, 0.1 M sodium phosphate, 0.01 M Tris-HCl (pH 8)). The suspension was repeatedly vortexed and stirred

continuously to aid protein solubilisation. Solubilised inclusion bodies were centrifuged (15 min, 12k rpm, SLA1500 rotor), to remove the insoluble fraction. The supernatant was applied to a home-poured column containing Ni-NTA resin (Qiagen) polypropylene column (Qiagen) and bound protein species were eluted with IMAC buffer B (500 mM imidazole, 0.02 M MOPS (pH 7)). recPrP-containing fractions were buffer-exchanged using a PD-10 desalting column (GE Healthcare) equilibrated with 20 mM sodium citrate (pH 3.4). The eluate was collected in 500 µl fractions and fractions containing the most recPrP were pooled. Protein concentrations at this stage were 1.87 and 1.56 mg/ml respectively for WT-recPrP and 101L-recPrP, and the concentration of WT-recPrP was adjusted to that of the 101L-recPrP by dilution in sodium citrate. The protein samples were incubated at 45 °C and 10 µl samples were analysed by size exclusion chromatography by use of a 300 x 7.8 nm TSKG4000SWxl HPLC column equilibrated with 20 mM sodium citrate (pH 3.4) at a flow rate of 1 ml/min. The eluate was monitored by UV absorbance at 280 nm. Overnight incubation in sodium citrate buffer allowed formation of oligomers, as shown by a shift to smaller elution volumes. After filtration for sterilisation, final concentrations of oligomeric preparations were 0.44 and 0.33 mg/ml for WT-recPrP and P101L-recPrP respectively.

Purification of PrP for refolding to amyloid fibrils

This protocol follows those published by Makarava et al and Breydo et al, which were the most up to date at the time that our fibrils were produced (Makarava N, Baskakov IV (2008) Expression and purification of full-length recombinant PrP of high purity. In: Hill AF (ed) Methods Mol Biol. Humana Press Inc, 999 Riverview Dr, Ste 208, Totowa, Nj 07512-1165 USA, City, pp 131-143; Breydo L, Makarava N, Baskakov IV (2008) Methods for conversion of prion protein into amyloid fibrils. In: Hill AF (ed) Methods Mol Biol. Humana Press Inc, 999 Riverview Dr, Ste 208, Totowa, Nj 07512-1165 USA, City, pp 105-115). Inclusion body pellets were resuspended in IMAC buffer A (8 M urea, 100 mM sodium phosphate, 10 mM tris-HCl pH 8 containing 10 mM reduced glutathione). PrP was purified using IMAC chromatography (Ni-NTA superflow, Qiagen) eluting components bound to the column with a linear gradient from 0 to 100 % IMAC buffer B (8 M urea, 100 mM sodium phosphate, 10mM tris-HCl pH 4.5 containing 10mM reduced glutathione). Protein-containing fractions, as determined by Coomasie-stained SDS-PAGE gels, were pooled and desalted by use of a Superdex 75 column (GE Healthcare) eluting protein with a solution containing 6 M urea, 0.1 M Tris-HCl, pH 7.5. Oxidised glutathione (50 mM) was added to protein containing fractions to a final concentration of 0.2 mM and EGTA to a final concentration of 5mM and the solution was left overnight to oxidise the single disulphide bond. Oxidised protein was purified further by reversed phase HPLC (in batches to avoid overloading the column) by dilution of the protein two-fold into RP-HPLC buffer A (0.1 % (v/v) trifluoroacetic acid in water). The protein was loaded onto a reversed phase column (214TP101522, Vydac) by use of a superloop (GE Healthcare) and bound protein was eluted by an increasing gradient of RP-HPLC buffer B (0.1 % (v/v) trifluoroacetic acid in acetonitrile). Monomeric, full length recombinant PrP eluted at ~35 % RP-HPLC buffer B and the fraction from the peak of the elution profile was lyophilised so as to avoid including other chemically modified species, which elute prior to or subsequent to this peak. Elutions from different HPLC batches were pooled prior to lyophilisation. Lyophilised protein was stored at -20 °C prior to use.

For fibrillisation, lyophilised recPrP was dissolved in 6 M guanidine HCl pH 6.0 at a concentration of 3 mg/ml. A fibrillisation reaction consisting of 2 M guanidine HCl, 10 mM thiourea, 120 µg/ml recPrP, 50 mM 2-(*N*-morpholino)ethanesulfonic acid (MES) pH 6.0, 10 µM thioflavin T was prepared. Reaction mixture (160 µl) was placed into individual wells of a 96 well plate and 3 Teflon spheres were added to aid mixing. The plate was shaken at 37 °C on a plate shaker. Aliquots were removed and assayed for fibril formation by thioflavin T fluorescence (excitation at 444 nm and emission at 485 nm) on a plate reader. The presence of fibrils was confirmed by both electron microscopy (see figure 1 in the main paper) and also by the presence of a 16 kDa band following maturation of the fibrils at 80 °C and digestion with proteinase K, as published previously by Breydo et al. Fibrils were dialysed into 50 mM sodium acetate, pH 5.5 for use. Final concentration of fibrils were 0.4 and 0.07 mg/ml respectively for WT-recPrP and P101L-recPrP.

WT-recPrP

IMAC:

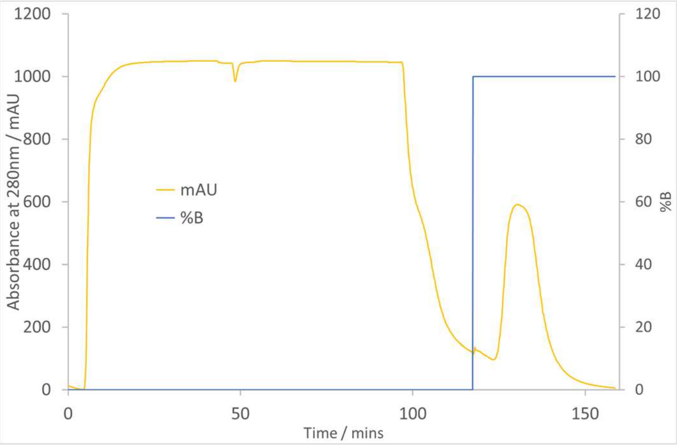

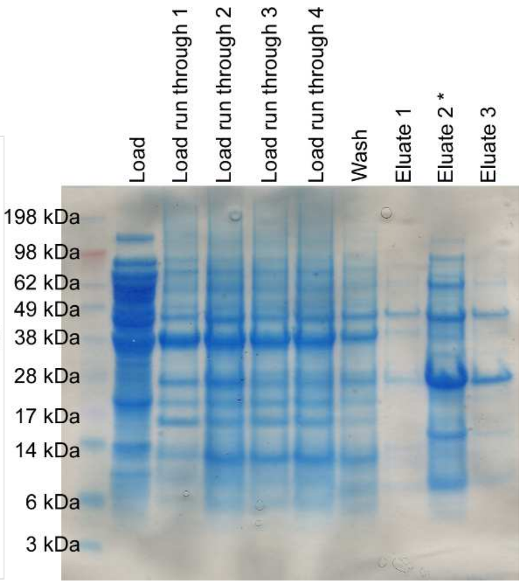

Cation exchange:

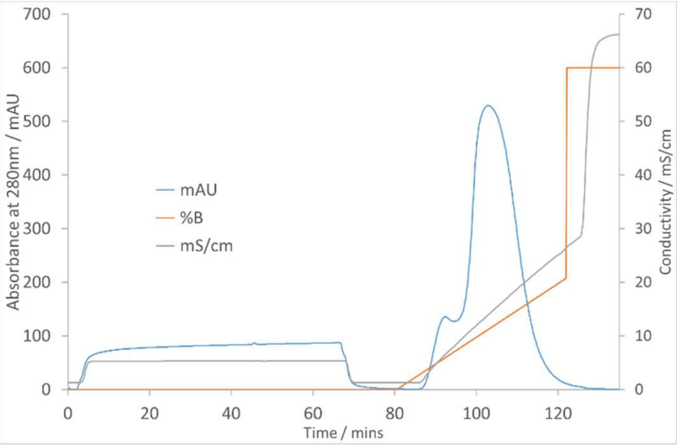

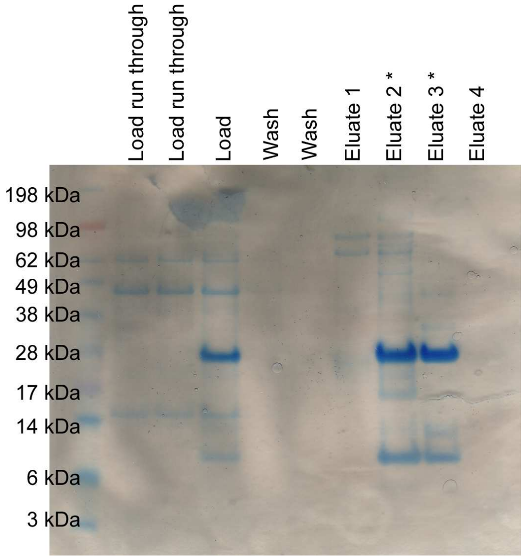

Circular dichroism:

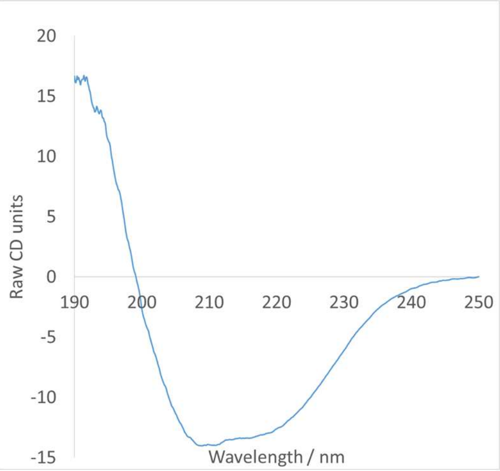

101L-recPrP

IMAC:

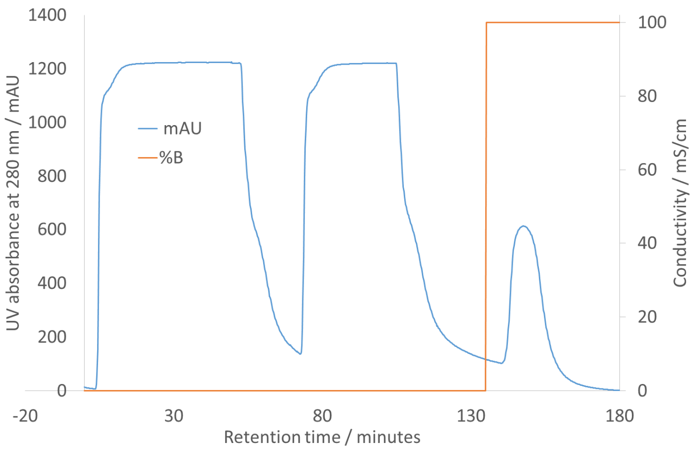

Cation exchange:

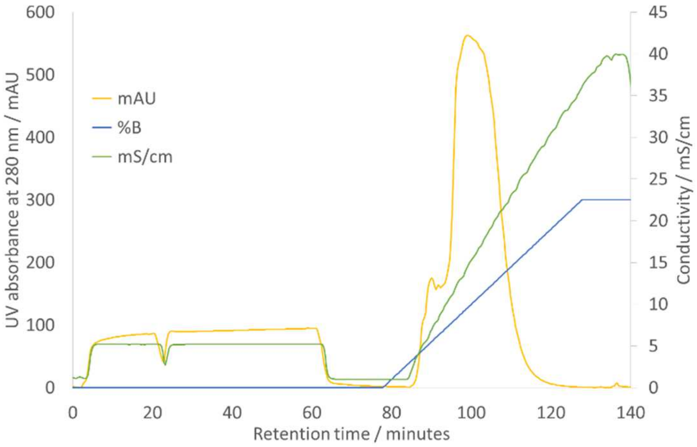

Circular dichroism

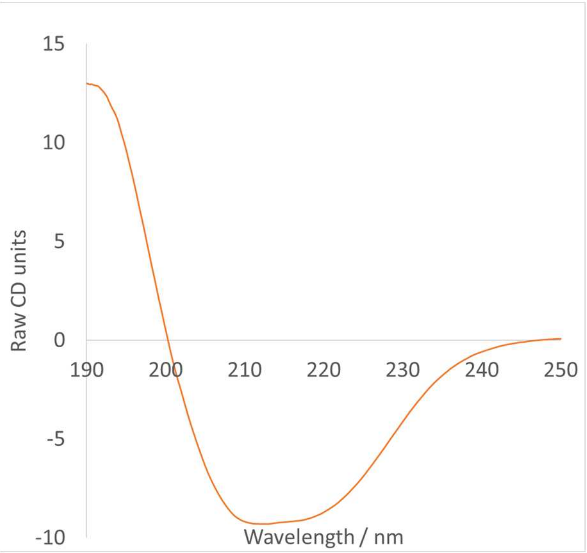

Oligomerisation:

WT-recPrP

IMAC:

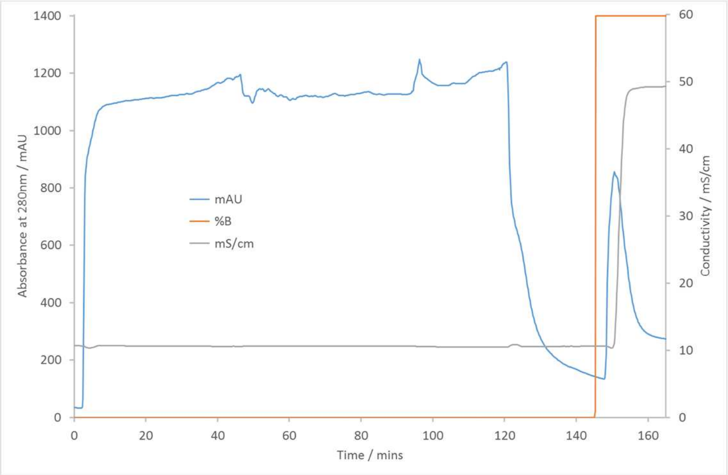

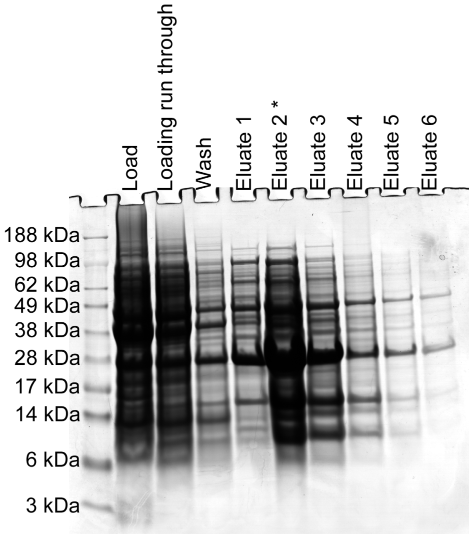

PD10 buffer exchange fractions:

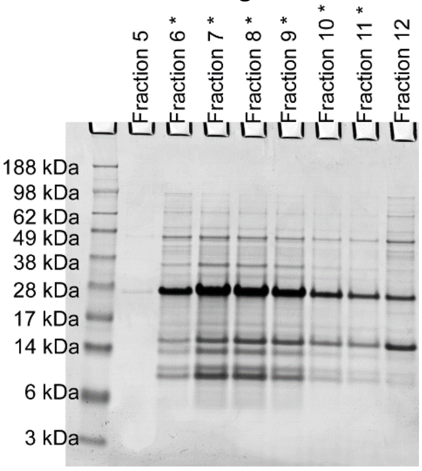

Oligomerisation:

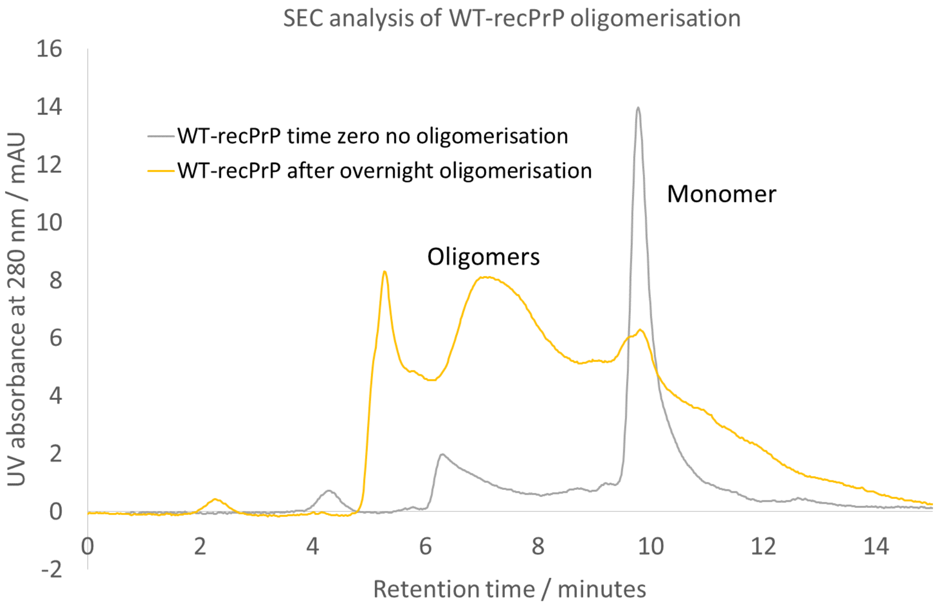

P101L-recPrP

IMAC:

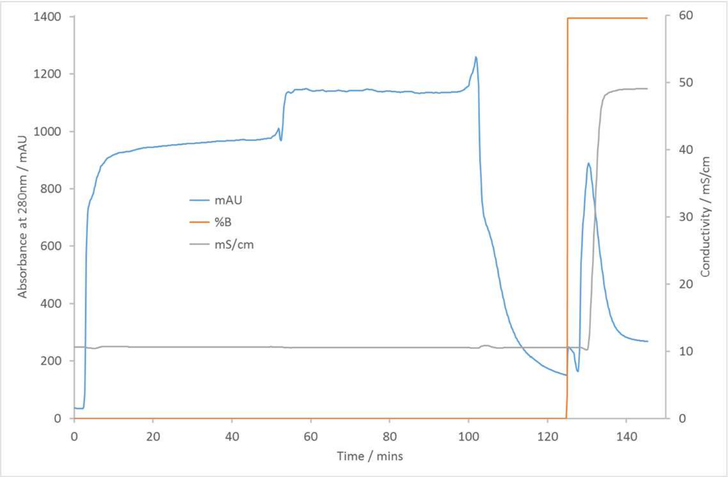

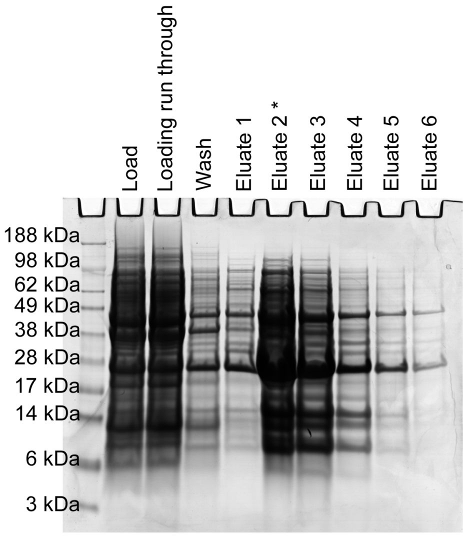

PD10 buffer exchange fractions:

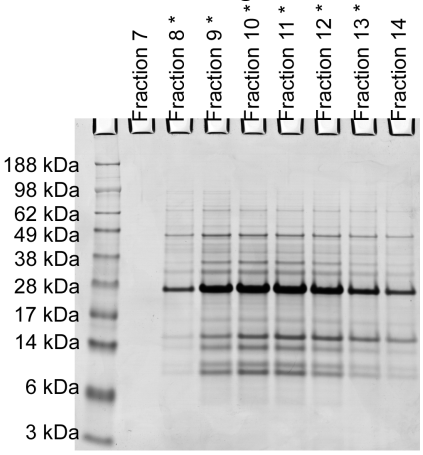

Oligomerisation:

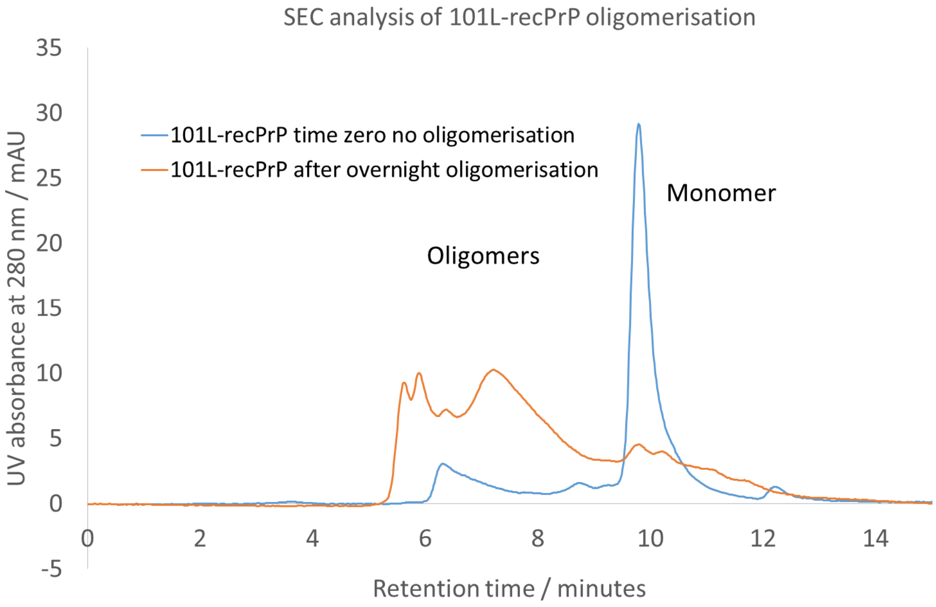

Purification for Fibrillisation

WT-recPrP

IMAC:

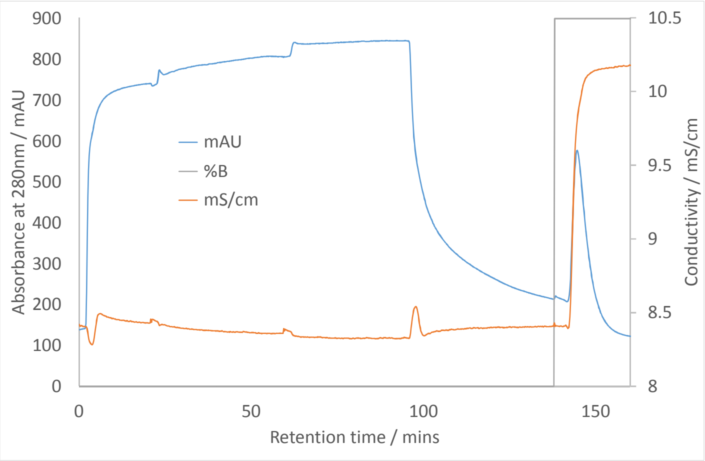

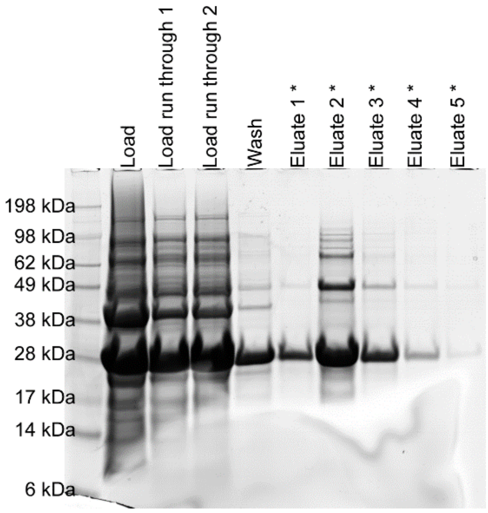

Desalting:

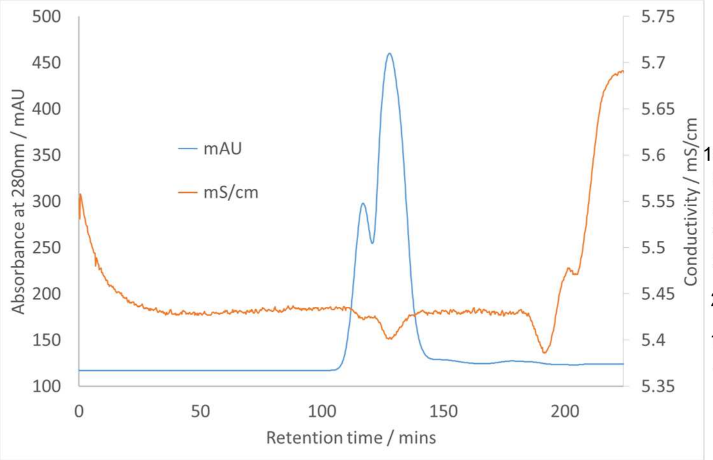

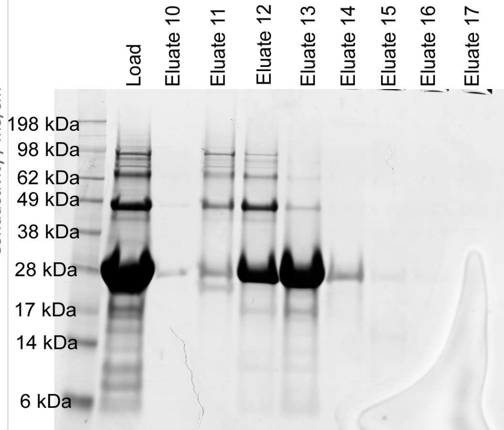

HPLC batch 1

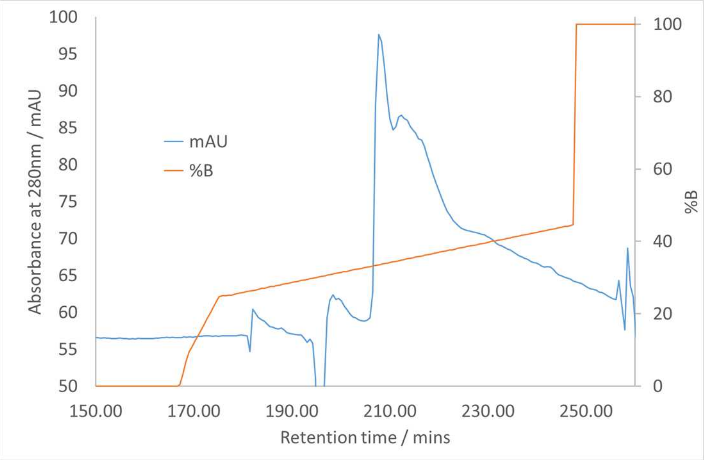

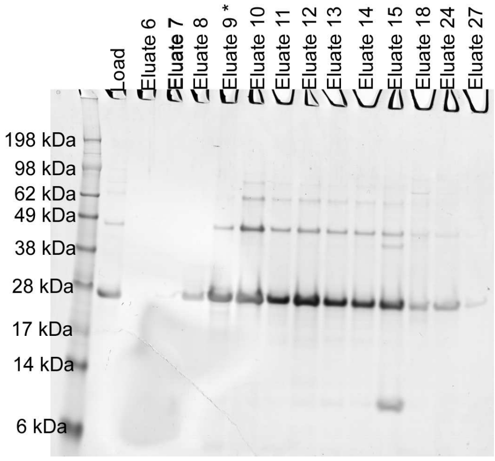

HLPC batch 2

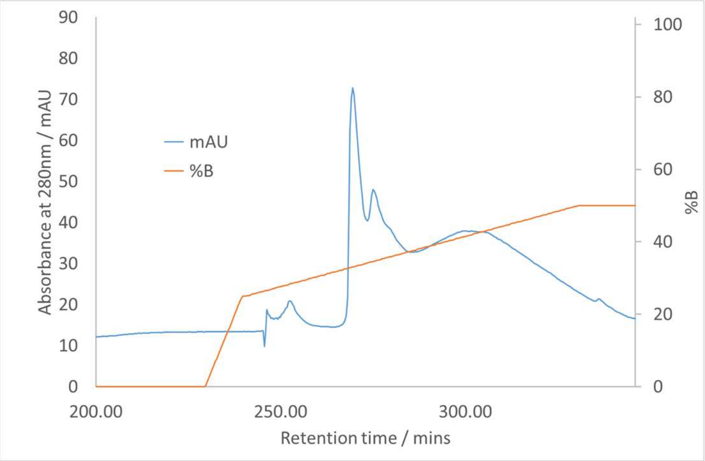

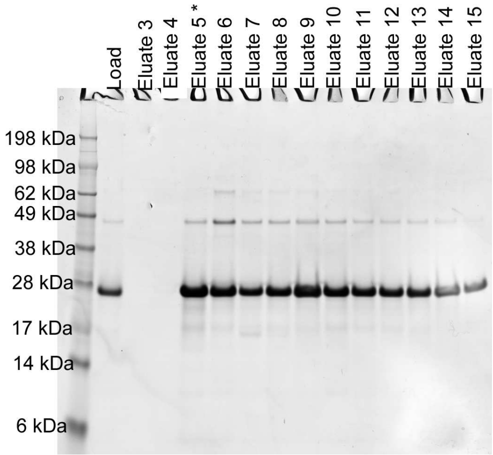

101L-recPrP

IMAC:

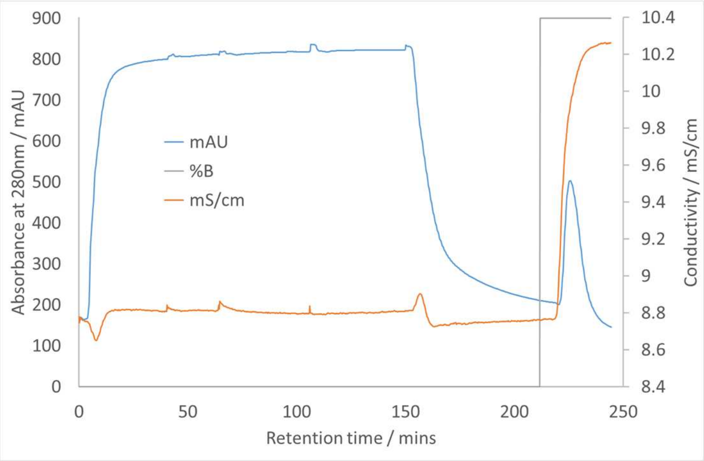

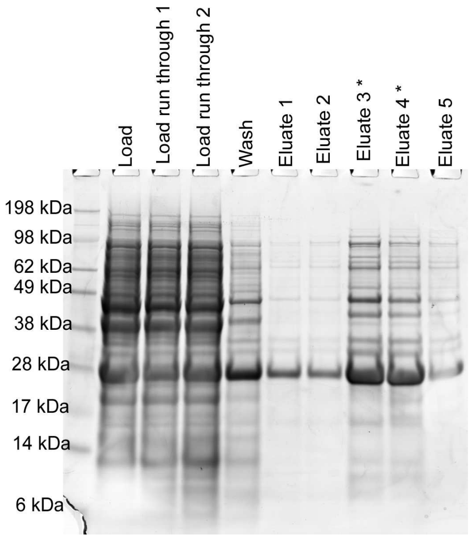

Desalting:

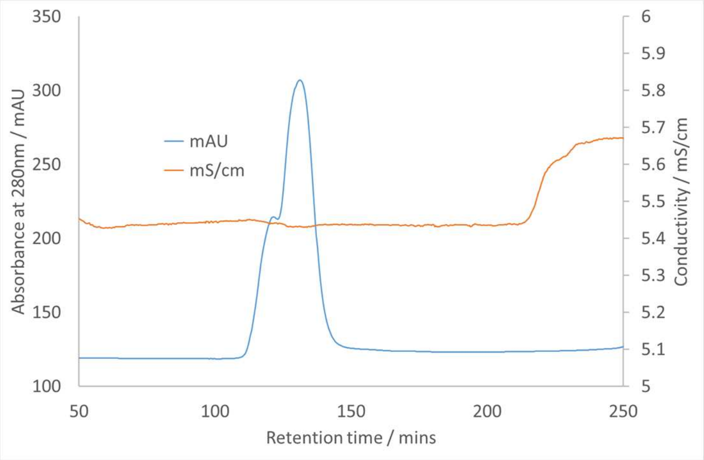

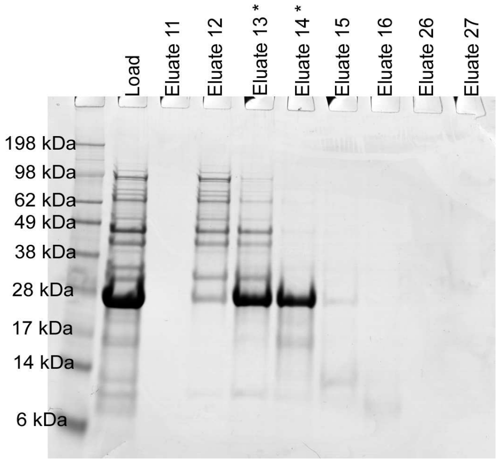

HPLC batch 1

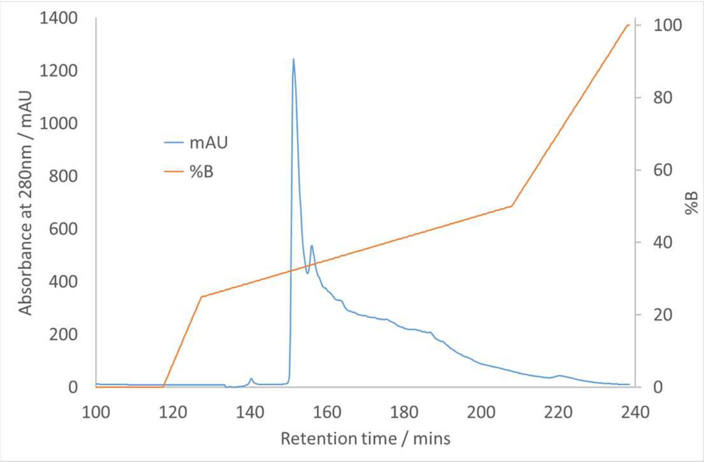

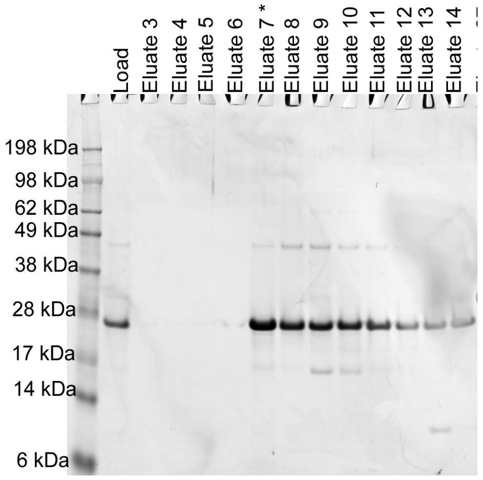

HPLC batch 2

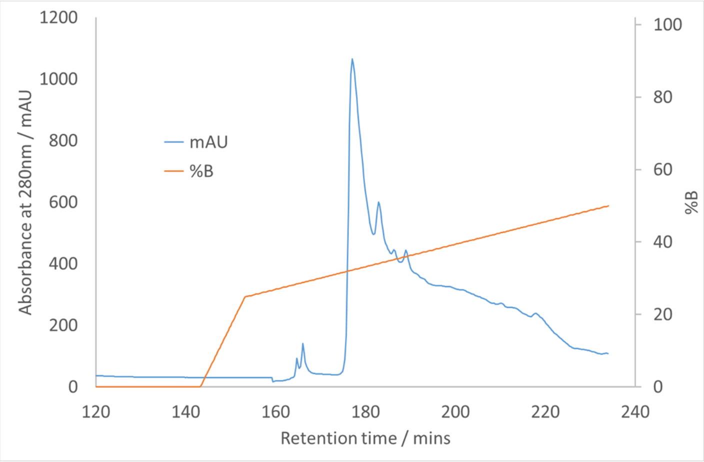

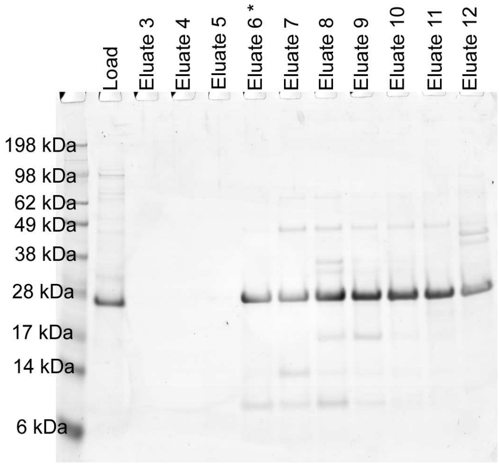

Fibrillisation

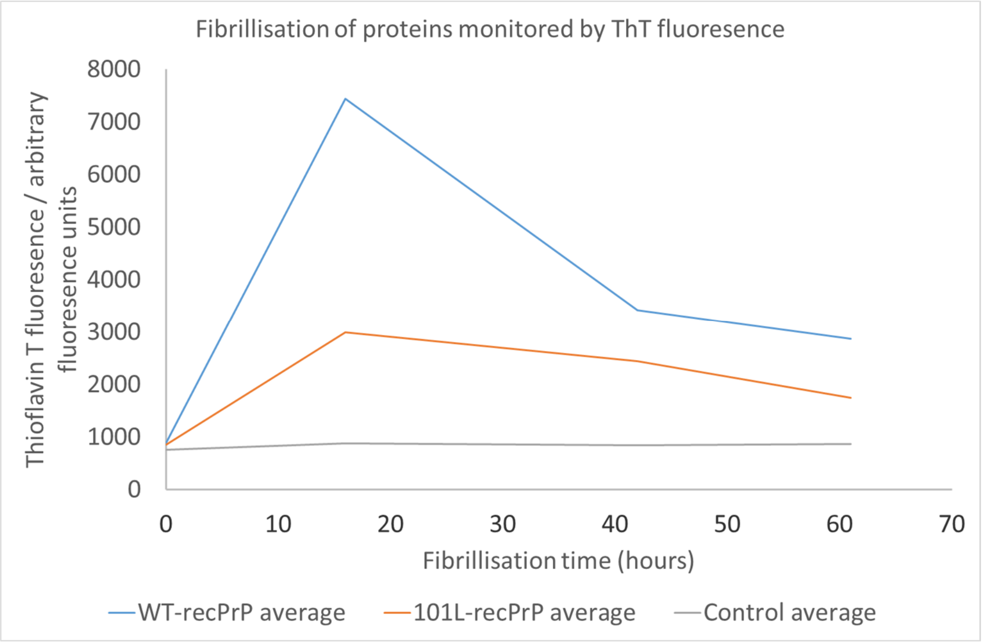

Silver stained gel of proteins after digestion with proteinase K either with or without maturation at 80 °C. The presence of a PK-resistant band at ~16 kDa is characteristic of PrP fibrils.

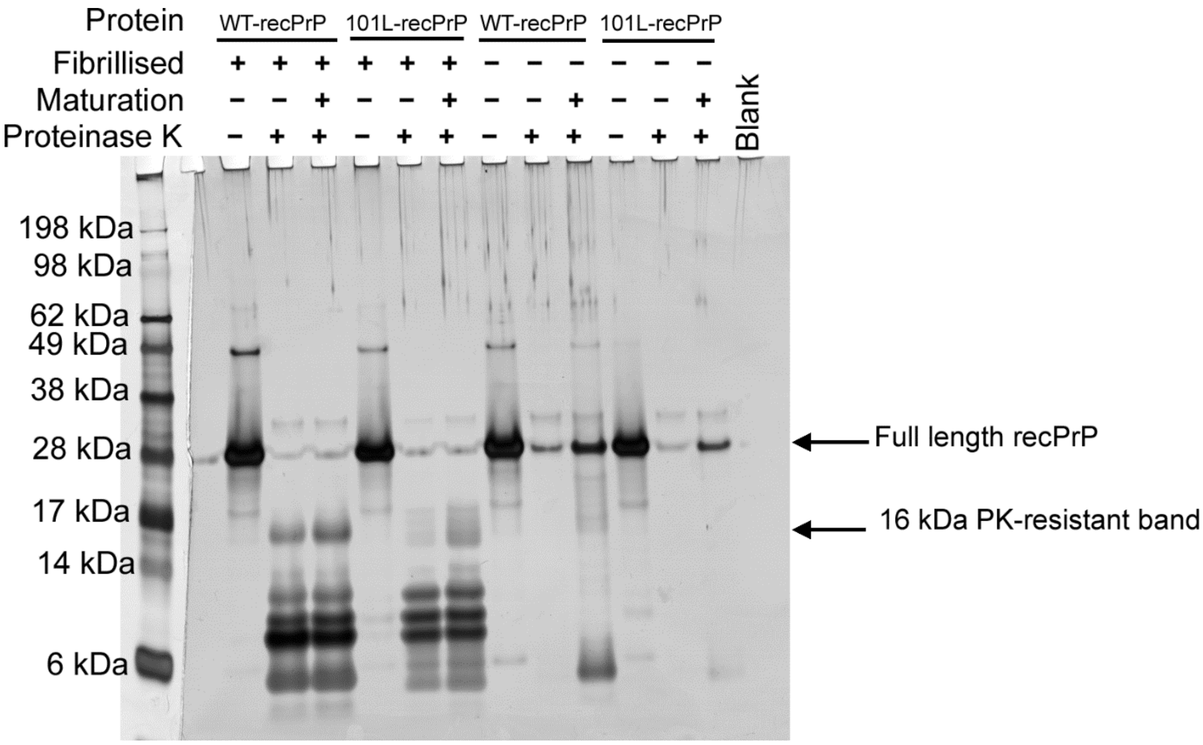

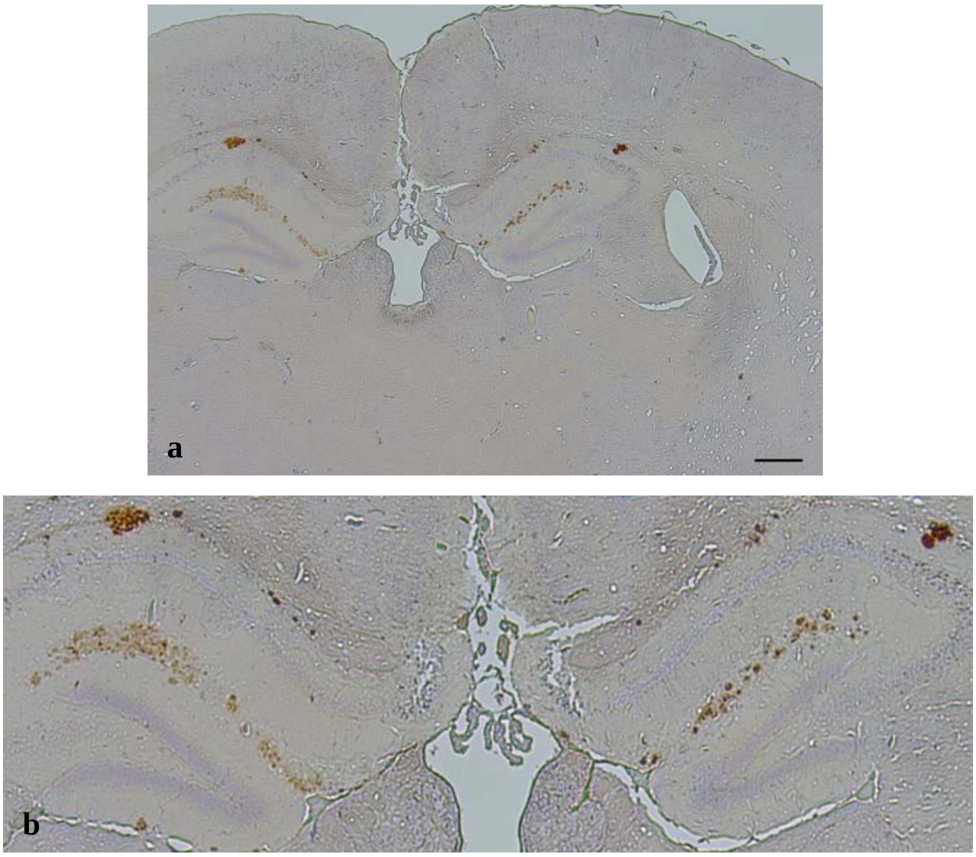

Supplementary Figure 1. Bilateral distribution of plaques

Immunohistochemical analysis of brain sections from a 101LL mouse inoculated with WT-recPrP fibril preparations. (a) Abundant plaque deposition was observed bilaterally and was most prominent in the corpus callosum and hippocampus. (b) Enlarged image. Section stained with anti-PrP Mab 6H4. Scale bar 200µm

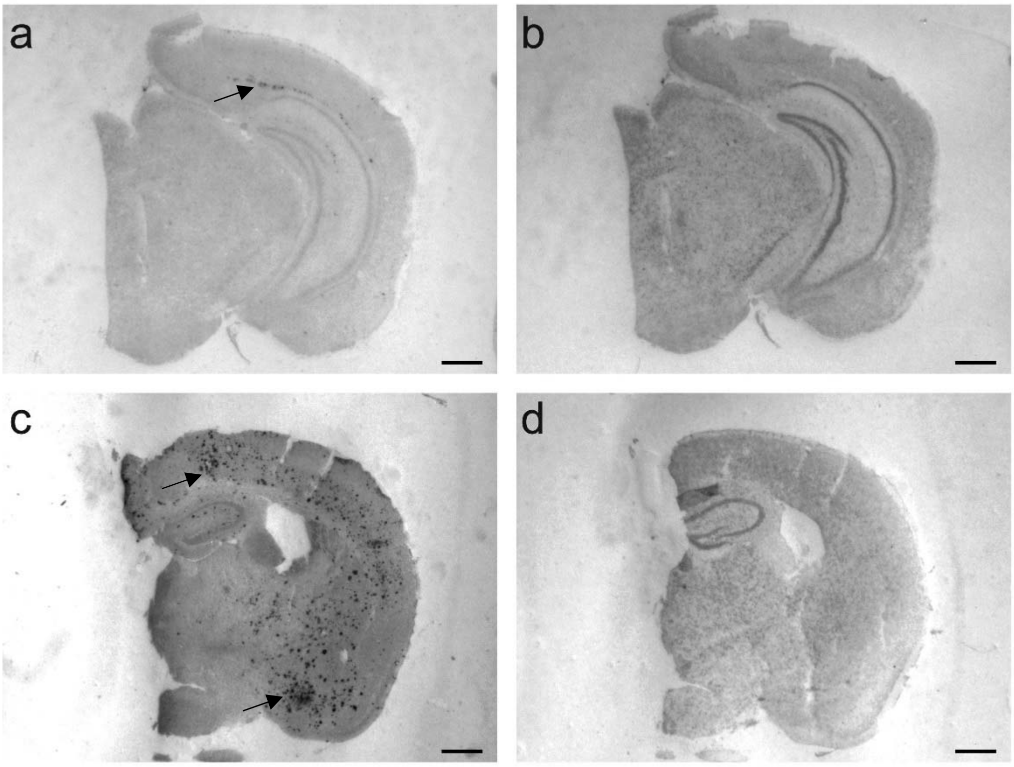

Supplementary Figure 2. PK resistance of PrP amyloid plaques.
Histoblots were performed as described in methods with overnight incubation in 20µg/ml proteinase K at 55ºC to identify PK resistant PrP. Blots were performed on tissue from a 101LL mouse inoculated with WT-recPrP, shown by IHC to contain PrP amyloid plaques. Tissue from a GSS22 transgenic mouse which overexpresses 101L PrP (Nazor et al 2005, EMBO J 24:2472) and has previously been shown to contain numerous PrP amyloid plaques (Piccardo et al 2013, J Virol 87:12349) was used as a positive control.
101LL mouse inoculated with WT-recPrP amyloid fibrils (a & b). GSS22 mouse overexpressing 101L PrP (c & d). Sections in a & c stained with anti-PrP Mab BH1, sections in b & d stained with normal mouse serum. PK-resistant amyloid plaques highlighted by arrows in a & c. Scale Bar 500µm.
